# Germline structural variation globally impacts the cancer transcriptome including disease-relevant genes

**DOI:** 10.1016/j.xcrm.2024.101446

**Published:** 2024-03-04

**Authors:** Fengju Chen, Yiqun Zhang, Fritz J. Sedlazeck, Chad J. Creighton

**Affiliations:** 1 Dan L. Duncan Comprehensive Cancer Center, Baylor College of Medicine, Houston, TX 77030, USA; 2 Human Genome Sequencing Center, Baylor College of Medicine, Houston, TX 77030, USA; 3 Department of Computer Science, Rice University, Houston, TX 77005, USA; 4 Department of Medicine, Baylor College of Medicine, Houston, TX 77030, USA

**Keywords:** cancer, structural variation, structural variants, germline, PCAWG, Pan-Cancer Analysis of Whole Genomes, Whole Genome Sequencing, GWAS, eQTL, expression quantitative trait loci

## Abstract

Germline variation and somatic alterations contribute to the molecular profile of cancers. We combine RNA with whole genome sequencing across 1,218 cancer patients to determine the extent germline structural variants (SVs) impact expression of nearby genes. For hundreds of genes, recurrent and common germline SV breakpoints within 100 kb associate with increased or decreased expression in tumors spanning various tissues of origin. A significant fraction of germline SV expression associations involves duplication of intergenic enhancers or 3′ UTR disruption. Genes altered by both somatic and germline SVs include *ATRX* and *CEBPA*. Genes essential in cancer cell lines include *BARD1* and *IRS2*. Genes with both expression and germline SV breakpoint patterns associated with patient survival include *GCLM*. Our results capture a class of phenotypic variation at work in the disease setting, including genes with cancer roles. Specific germline SVs represent potential cancer risk variants for genetic testing, including those involving genes with targeting implications.

## Highlights

• Combined RNA and germline SV analysis using the PCAWG consortium datasets
• Several genes impacted by germline SVs could conceivably contribute to cancer
• Several genes are essential in cell lines or involve patient survival associations

## Graphical abstract

**Figure:**
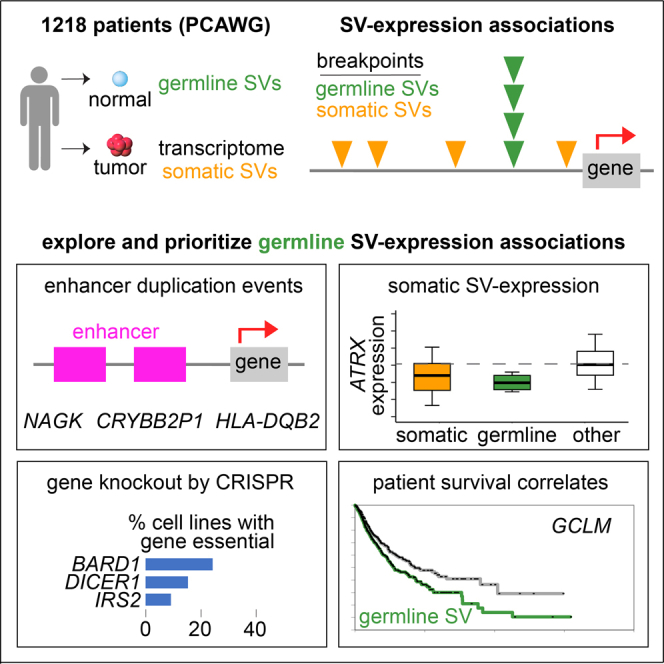

## Teaser

Analyzing tumor gene expression data and whole genome sequencing from the PCAWG consortium, representing 1,218 patients, Chen et al. identify genes with expression influenced by germline structural variation across diverse tissues. These findings have implications for establishing a class of germline variants associated with cancer risk.

## Introduction

Structural variation is a broad class of chromosomal variation that includes copy number variants (deletions and duplications), balanced rearrangements (e.g., inversions and translocations), and insertions (e.g., from mobile-elements). In recent years, structural variants (SVs) have been associated with an increasing number of normal phenotypic variations, as well as common and rare human diseases.^1^^,^^2^ Recent technological advances, including higher resolution microarrays and massively parallel next-generation sequencing, have allowed for more accurate cataloging of structural variation across many thousands of individuals.^3^ Cancer is a multistep process involving mutations in multiple genes, to which germline mutations may contribute and provide a head start on the neoplastic process.^4^ Moderate-to high-penetrance germline variants in cancer predisposition genes underlie 5%–10% of all cancers, with SVs representing an uncommon cause of cancer susceptibility, underlying perhaps 1.5% of cancer cases by some estimates.^5^ SV-impacted genes in cancer would include cancer predisposition genes, DNA damage response genes, and somatic driver genes.^5^^,^^6^ The functional effects of germline SVs on gene expression in normal tissues or cell lines derived from normal tissues have been explored,^7^^,^^8^^,^^9^^,^^10^^,^^11^ whereby SVs can have large effect sizes on adjacent genes and are often constitutive across diverse tissues.^7^^,^^10^ At the same time, the impact of germline SVs on expression variation in human cancers would also be of interest.

Both germline variation and somatic alterations contribute to the molecular profile of cancers. Somatic structural variation exerts a strong influence on gene expression in cancer.^12^^,^^13^^,^^14^^,^^15^^,^^16^ Recently, the Pan-Cancer Analysis of Whole Genomes (PCAWG) consortium aggregated whole genome sequencing (WGS) data from 2,658 cancers across 38 tumor types involving 20 major tissues of origin to identify germline variants and somatically acquired mutations.^6^ In the PCAWG cohort, integration of somatic SVs with corresponding RNA data identified genes with altered expression associated with nearby SV breakpoints.^14^^,^^16^^,^^17^ Genes deregulated or disrupted in this way included many oncogenes, such as *TERT*, *MDM2*, *CDK4*, *ERBB2*, *CD274*, *PDCD1LG2*, *BCL2*, and *IGF2*; and tumor suppressor genes, such as *PTEN*, *RB1*, *STK11*, and *TP53*. In the PCAWG-led studies, common and rare germline variants were found to affect somatic mutation patterns, including SVs.^6^^,^^18^ Germline deletion SVs impacting cancer susceptibility genes were cataloged in the PCAWG cohort, e.g., involving *BRCA1* and *BRCA2*. However, the above studies did not systematically explore the potential for germline SVs, including SVs with breakpoints outside of genes, to impact gene expression, as was explored for the somatic SVs.

In the present study, we combined patient germline SV data (taken from a normal blood sample) with tumor RNA sequencing (RNA-seq) data across the PCAWG cohort to systematically catalog gene-level associations with altered tumor expression in conjunction with nearby germline SV breakpoints. We took a gene-centric approach to the data, allowing us to focus on genes of interest that were recurrently altered in relation to SVs. The germline SV-expression associations identified cut across tumors from various tissues of origin. Most of the significant genes would not necessarily have specific roles in cancer, but would instead reflect germline variations. However, some genes had known roles in cancer, seemed to be targeted by somatic SVs, were essential in cancer cell lines, or had germline patterns associated with cancer patient survival.

## Results

### Germline structural variation patterns across cancer patients

**Figure 1:**
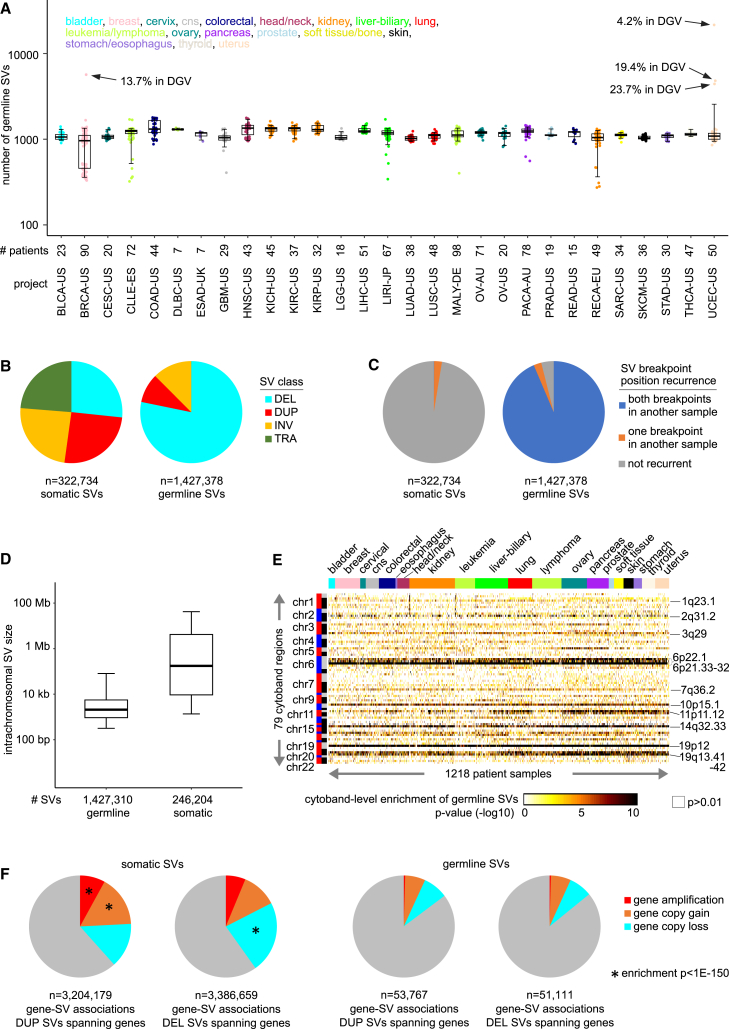
Germline structural variation patterns in the PCAWG patient cohort (A and B) By TCGA or ICGC project, boxplot of the total numbers of germline SVs detected in the matched blood normal for each tumor sample, involving 1,218 patients with combined data by WGS (both tumor and normal) and RNA-seq (tumor only). Four outlier patients with very high numbers of SVs in the blood are indicated, each having a smaller percentage of SVs represented in the DGV^3^. For the other patients, 54%–92% of germline SVs (based on blood) were in DGV (Table S1, Figure S1C), (B) SV class distribution, as observed for the 1,427,378 germline SVs and the 322,734 somatic SVs identified for the 1,218 patient samples. DEL, deletion; DUP, duplication; INV, inversion; TRA, translocation. (C) SV breakpoint position recurrence, as observed for the germline SVs and for the somatic SVs identified for the 1,218 patient samples. Pie charts indicate percentages of SVs with one or both breakpoints represented in another SV found for a different patient sample (allowing 10 bp of slop). (D) Boxplot of the intrachromosomal SV size for the germline SVs as compared with the somatic SVs. (E) Top 79 cytoband regions (out of 811, not considering X or Y chromosomes) enriched for SV events, with p < 0.0001 (chi-squared test) for at least 20 of the 1,218 patient samples. (F) For both germline SV and somatic SV call sets, gene-SV associations with SV breakpoints spanning the gene for a specific sample are broken down by gene-level copy number (corrected for tumor ploidy; amplification, >5 copies; copy gain, 3–5 copies; copy loss, 0–1 copies), for duplication SVs versus deletion SVs. Enrichment patterns involving somatic but not germline SVs (by chi-squared test) are indicated. For (A) and (D), boxplots represent 5%, 25%, 50%, 75%, and 95%.

To explore germline structural variation in cancer, we referred to the PCAWG dataset of germline and somatic SVs calls representing 1,218 patients (Table S1). Based on the blood normal sample, a median of 1,163 germline SVs was identified per patient (with s SD of 650.4). On average, the numbers of germline SVs detected did not vary widely according to tumor tissue origin (Figure 1A). However, four patients had very high numbers of SVs detected in their blood sample, only a fraction of which constituted previously identified SVs. The four patients (one breast cancer, three uterine cancers) might conceivably have undergone clonal hematopoiesis,^19^ although the SVs unique to these patients did not contribute to the SV-expression associations described below (Table S2). Across patients, we observed no strong associations between germline SV numbers and patient age, tumor ploidy, or tumor cellularity (Figure S1A). In contrast with the somatic SVs, most germline SVs in the PCAWG dataset were deletions (78%), followed by inversions and duplications (12.5% and 9.3%, respectively) (Figure 1B). Of 1,427,378 total germline SVs in the PCAWG dataset, only 68 were inter-chromosomal translocations, and 24% of the 322,734 PCAWG somatic SVs were translocations. In addition, germline SVs were highly recurrent across patients in terms of breakpoint positions, with more than 93% of germline SV calls involving SVs with both breakpoints found for two or more patients, while this applied to only 0.1% of somatic SV calls (Figure 1C). For intra-chromosomal SVs, the median distance between breakpoints was much smaller for germline versus somatic SVs, 2.1 kb versus 175 kb, respectively (Figure 1D). In addition, the numbers of somatic SVs detected per tumor varied widely across patients (Figure S1B), and an increased total number of somatic SVs was significantly associated with higher patient age (Spearman’s r = 0.13; p < 1E−5) and strongly associated with higher tumor ploidy (r = 0.37; p < 1E−40).

Most of the germline SVs in the PCAWG dataset had been observed elsewhere, with 85% of these SVs being represented in the Database of Genomic Variants (DGV) curated from published studies,^3^ while only ∼0.1% of PCAWG somatic SVs were similarly represented in DGV (Figure S1C, Table S2). Germline SV events tended to be more frequent within specific genomic regions across patients. For each patient, we assessed cytoband-level enrichment of germline SVs, with a top set of 79 cytoband regions—including 1q23.1, 3q29, 6p22.1, 6p21.33–32, 14q32.33, 19p12, and 19q13.41–42—identified as significant (p < 0.0001 by chi-squared test) for at least 20 patients (Figures 1E and S1D, Table S1). Duplication and deletion SVs with breakpoints spanning genes should be reflected in the corresponding gene copy number. However, for gene copy number levels as measured in the PCAWG tumor samples, somatic SVs but not germline SVs showed the anticipated relationships involving duplications and deletions (Figure 1F). This result is likely because tumors exhibit extensive somatic copy number alterations in association with somatic SVs^15^—e.g., as reflected in the relationship between somatic SVs with tumor ploidy (Figure S1B)—which would overshadow any germline copy number variants. The PCAWG germline SV call set used here should not include mobile-element insertions (MEIs), most of which would be inactive and unable to retrotranspose, though a subset of MEIs would be somatically active in cancer.^20^

### Gene-level SV-associated gene expression alterations

We set out to identify the genes for which the proximity of germline SVs was recurrently and significantly associated with altered expression across multiple patients. Using integration approaches between SVs and gene expression, previously demonstrated in tumors for somatic SVs,^14^^,^^15^^,^^17^^,^^21^^,^^22^^,^^23^^,^^24^ we assessed gene-level associations between tumor expression and nearby germline SV breakpoints across the PCAWG tumor cohort. As observed for somatic SVs, germline SVs with breakpoints near a gene could lead to altered *cis*-regulation (e.g., involving enhancer duplication) or gene disruption (e.g., for breakpoints falling within the gene). For each gene with expression data from the tumor, we assessed the pattern of nearby germline SV breakpoints within a given region window (e.g., 100 kb upstream of the gene). From the PCAWG data, we assembled a data matrix of breakpoint patterns for all 22,956 named genes and 1,218 tumors. We then assessed the association between expression and germline SV breakpoint pattern for each gene by linear models correcting for covariates, including tumor tissue of origin. In some respects, our overall approach to associate SVs with expression parallels the concept of expression quantitative trait loci (eQTLs), but with our approach being gene relative and region specific rather than SV specific.

**Figure 2:**
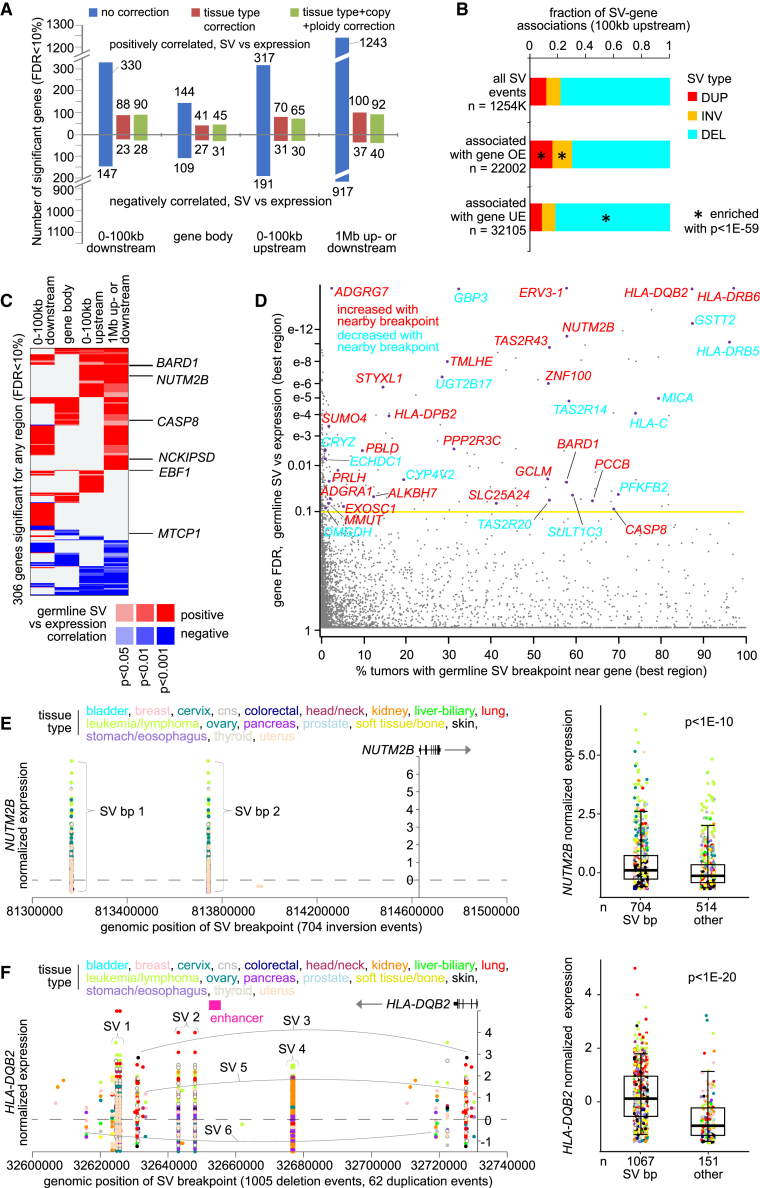
Genes with altered mRNA expression recurrently associated with nearby germline SV breakpoints (A) For each of four genomic region windows in relation to genes (100 kb upstream of the gene, 100 kb downstream of the gene, within the gene body, or 1 Mb upstream or downstream of the gene), the numbers of significant genes (using a FDR of <10%) showing the association between mRNA expression and nearby germline SV breakpoint. Numbers above and below the zero point of the y axis denote positively and negatively correlated genes, respectively. Linear regression models evaluated significant associations with and without corrections for specific covariates, as indicated. (B) Breakdown by SV class (duplication, inversion, deletion) for the germline SV-gene associations with breakpoints 100 kb upstream of the gene involving increased or decreased expression, respectively (p < 0.01 and expression greater than or less than the tumor median, respectively). Enrichment p values by chi-squared test. (C) Heatmap of significance patterns for 306 genes associated with altered expression in conjunction with nearby germline SV breakpoints (FDR of <10%, after correction for covariates), for any genomic region window examined. Genes listed are cancer-associated by COSMIC.^25^ (D) Significance of germline SV-impacted genes at the mRNA level (best gene FDR among the genomic region windows 100 kb upstream of the gene, 100 kb downstream, and within the gene), as plotted (y axis) versus the percentage of samples with a germline SV breakpoint in the genomic region window (best gene FDR) relative to the gene impacted (x axis). (E) *NUTM2B* mRNA levels in tumors corresponding to germline SV breakpoints located in the genomic region 100 kb upstream of the gene (left). Each SV for a given patient sample has two breakpoints represented. Boxplot (right) shows *NUTM2B* expression by patient samples with or without SV breakpoint 100 kb upstream of gene. (F) Similar to (E), but for germline SV breakpoints located downstream of *HLA-DQB2*. For (B–D), SV-expression association p values and FDRs correct for tissue type (by TCGA or ICGC project), gene-level copy, tumor ploidy, and tumor purity by linear modeling. For (E and F), differential expression p values correct for tissue type (using the categories represented in the figure), and boxplots represent 5%, 25%, 50%, 75%, and 95%.

By integrating transcriptomic data (from tumors) with germline SV data^23^ (from blood normal), hundreds of genes showed significantly altered gene expression in relation to nearby germline SV breakpoints (relative to tumors without breakpoints). SV breakpoints associated with altered expression include breakpoints located downstream or upstream of genes or within the gene boundaries (Figure 2A, Table S3). For regions 100 kb upstream of the gene, 100 kb downstream of the gene, within the gene body, or 1 Mb upstream or downstream of the gene, the numbers of significant genes at a false discovery rate (FDR) of less than 10% were 95, 119, 76, and 132, respectively, after correction for tumor tissue of origin, tumor ploidy, tumor purity, and gene-level copy number. For each gene set, more genes were positively correlated with SV event (i.e., expression was higher when SV breakpoint was present) than were negatively correlated. Without correcting for tissue or origin, we found even greater numbers of genes with germline SVs associated with increased expression (Figure 2A), indicating that the tissue of origin versus the SV breakpoint patterns would better explain the differential patterns observed for those additional genes. Unlike what has been observed for the somatic SVs,^14^^,^^15^ incorporating gene-level copy number and tumor ploidy did not greatly impact the germline SV-expression associations observed. All major SV classes (duplication, inversion, and deletion) were involved in the significant germline SV-expression associations. However, associations with increased expression were significantly enriched for duplication and inversion SVs, while associations with decreased expression were enriched for deletion SVs (Figure 2B). A set of 306 genes associated with altered expression in conjunction with nearby germline SV breakpoints (FDR of <10%, after correction for tissue type, gene-level copy, tumor ploidy, and tumor purity) for any of the above four genomic region windows examined (Figures 2C and 2D).

Genes with differential expression significantly associated with nearby germline SV breakpoints could each involve a substantial percentage of patients with SVs. For some significant genes, as few as ∼2% of patients could have an SV breakpoint in the associated genomic region, but for other genes, more than 80% of patients may be similarly involved (Figure 2D). Examples of significant genes, including *NUTM2B* and major histocompatibility complex, class II, DQ beta 2 (*HLA-DQB2*), highlight breakpoint patterns for highly recurrent germline SVs involving increased mRNA levels (Figures 2E and 2F). Germline SVs with breakpoints 100 kb upstream of NUT family member 2B (*NUTM2B*) involved a recurrent inversion event with a size of ∼58 kb in 704 patients (Figure 2E; Table S4). Germline SVs with breakpoints 100 kb downstream of *HLA-DQB2* involved multiple recurrent SVs and both deletion events and duplication events, with breakpoints in 1,067 patients (Figure 2F; Table S4). Notably, all 62 duplication events downstream of *HLA-DQB2* spanned an enhancer element. For genes such as *NUTM2B*, the absolute expression differences between SV-associated and other patients may be relatively small, but still statistically significant, even when correcting for expression differences by tumor tissue of origin (Figure 2E). We also found our germline SV-expression associations to share significant overlaps with SV-eQTLs previously cataloged using normal tissues from the Genotype-Tissue Expression project^7^ (Figures S2A and S2B). A significant number of our SV-expression associations were also present in a breast cancer cell line^26^ (Figure S2C). Our germline SV-expression associations were not confounded by somatic SV or germline single nucleotide variants (SNVs) patterns (Figure S3A).

In contrast with previous observations involving somatic SVs,^14^ incorporating tumor tissue of origin into the linear modeling of germline SV breakpoints versus gene expression resulted in a substantial decrease in the numbers of significant genes (Figure 2A). This result suggests that some germline SVs would be more represented in patients with specific cancer types, as the tissue type over the SV breakpoint pattern would better explain the differential expression observed. A top set of recurrent germline SVs specific to tumor tissue of origin showed significant enrichment, predominately in patients with leukemia, kidney cancer, head and neck cancer, or liver cancer, with some SVs more enriched in other tissues (Figure S3B). As opposed to the genes significant both with and without correction for tumor tissue of origin, most of the genes significant only for the model without tissue type correction had both breakpoint enrichment pattern and corresponding differential expression for at least one tissue type (Figures S3C and S3D). All SV-expression associations explored below incorporate tumor tissue type, gene-level copy, tumor ploidy, and tumor purity, where the associations would span tumors from multiple tissues of origin. However, recurrent germline SVs enriched by tumor tissue type may warrant further attention elsewhere, e.g., as part of genome-wide association studies (GWASs). Few genes were significant when assessing a 2-kb upstream genomic region window for germline SV-expression associations (Table S3).

### Germline SV-associated expression involving enhancers or 3′ UTRs

**Figure 3:**
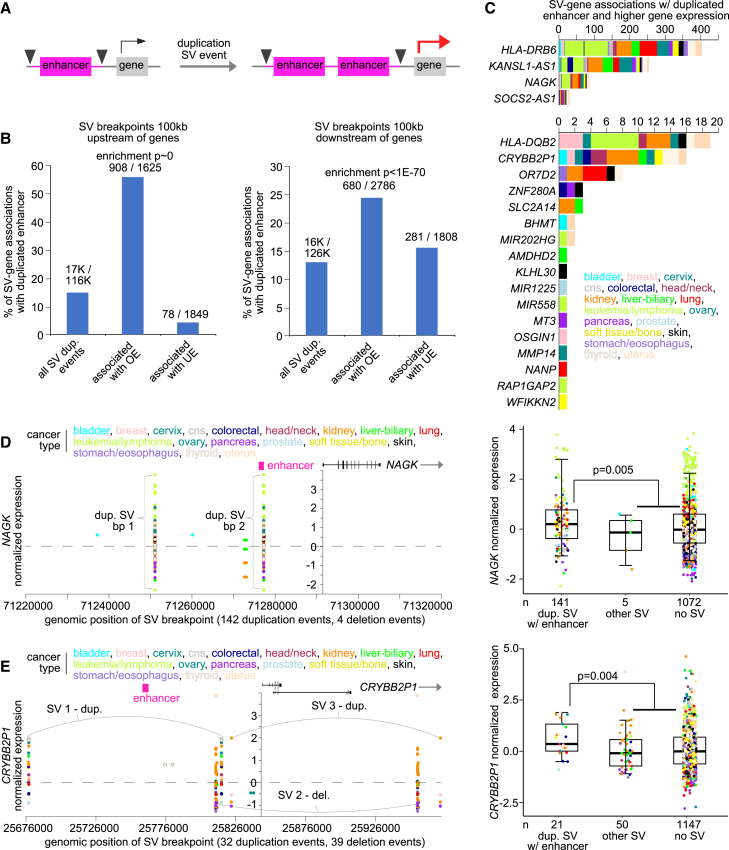
Germline duplication of intergenic enhancer regions is associated with higher expression of nearby genes (A) Schematic of the phenomenon of interest. Germline duplication SVs with breakpoints spanning an enhancer region may result in higher expression of nearby genes. (B) Percentages of germline SV-gene associations with SV breakpoints outside of the gene but spanning an enhancer region,^28^ as tabulated for the entire set of SV-gene associations 100 kb upstream (left) or 100 kb downstream (right), as well as for the subsets of associations involving altered gene expression (p < 0.01 by linear modeling). Enrichment p values by chi-squared test. (C) By gene and by tumor tissue of origin, the number of germline SV breakpoint associations involving the duplication of an enhancer with increased gene expression in the tumor sample (gene-level p < 0.01 across samples by linear modeling for either 100 kb upstream or 100 kb downstream regions, with expression in tumor greater than the sample median), representing 21 genes and 634 patients. (D) *NAGK* mRNA levels in tumors corresponding with germline SV breakpoints located in the genomic region 100 kb upstream of the gene and spanning an enhancer region (left). Each SV for a given patient sample has two breakpoints represented. Boxplot (right) shows *NAGK* expression by patient samples with enhancer-spanning SVs within 100 kb upstream of gene start versus samples with other upstream SVs not enhancer-associated versus other patient samples. (E) Similar to (D), but for germline enhancer-associated SV breakpoints located upstream of *CRYBB2P1*. For (B and C), SV-expression association p values and FDRs correct for tissue type (by TCGA or ICGC project), gene-level copy, tumor ploidy, and tumor purity by linear modeling. For (D and E), differential expression p values correct for tissue type (tissue type categories as provided in the same figure), and boxplots represent 5%, 25%, 50%, 75%, and 95%. Duplication SVs spanning an enhancer region, but either spanning the gene or having breakpoints within the gene are not represented in the above results.

Various mechanisms could be at work in germline SV-associated altered expression.^17^ One mechanism evident in the PCAWG datasets involved the duplication of intergenic enhancer regions (Figure 3A).^27^ In the PCAWG datasets, we cataloged germline duplication SV events with breakpoints 100 kb upstream or 100 kb downstream of a gene, where the SV breakpoints did not fall within the gene nor span the gene. As compared with all genes with associated SVs, the subset of genes with over-expression—but not under-expression—in conjunction with nearby germline SV breakpoints (p < 0.01 by linear model correcting for covariates) were highly enriched (p < 1E−70, chi-squared test) for SVs associated with enhancer duplication (Figure 3B). Genes involved in enhancer duplication events combined with higher expression included *HLA-DRB6*, *KANSL1-AS1*, *NAGK*, *SOCS2-AS1*, *HLA-DQB2*, crystallin beta B2 pseudogene 1 (*CRYBB2P1*), and *OR7D2* (Figure 3C), with the associations spanning various tissues. *HLA-DQB2*, noted above (Figure 2F), involved both duplication SVs spanning a downstream enhancer and deletion SVs not spanning the enhancer, with both SV groups being associated with higher *HLA-DQB2* expression. *NAGK* (N-acetylglucosamine kinase) involved 141 patients with a recurrent duplication SV with breakpoints upstream of the gene and spanning an enhancer element, while other SV deletion events were not associated with higher expression (Figure 3D). Similarly, *CRYBB2P1* involved 32 duplication SV events—21 spanning an upstream enhancer—and 39 deletion events, where only the enhancer-spanning duplication SVs were associated with higher expression (Figure 3E).

**Figure 4:**
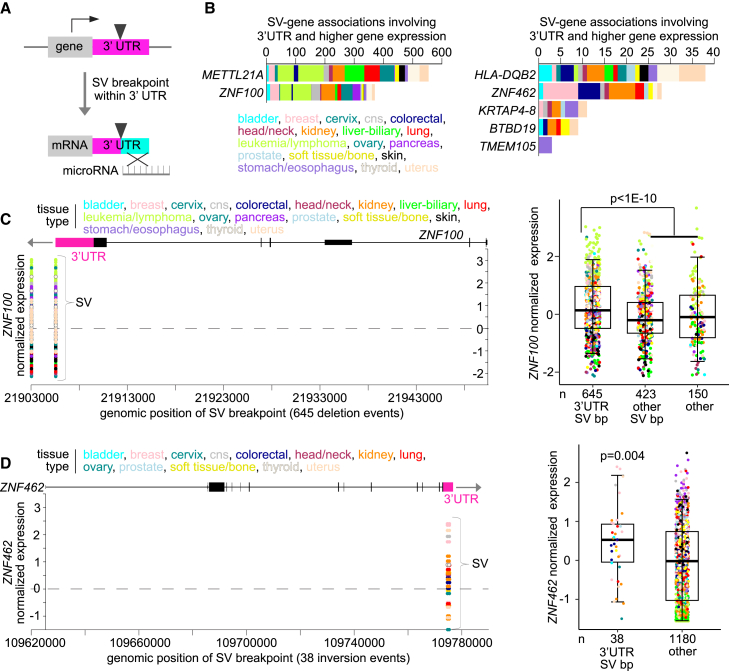
Disruption of 3′ untranslated regions (UTRs) by germline SVs associated with higher gene expression (A) Schematic of the phenomenon of interest. Germline SVs with breakpoints falling within a gene 3′ UTR may result in higher gene expression, e.g., through disrupting microRNA-mediated mRNA regulation or regulatory protein binding. (B) Significant genes (using a FDR of ≤10%) showing the association between higher gene expression in the tumor tissue sample and germline SV breakpoint falling within the gene 3′ UTR, involving 7 genes and 780 patients with expression greater than the tumor sample median. (C) *ZNF100* mRNA levels in tumor samples corresponding with germline SV breakpoints located in the gene 3′ UTR (left). Each SV for a given patient sample has two breakpoints represented. Boxplot (right) shows *ZNF100* expression by patient samples with SV breakpoint within the gene 3′ UTR versus other patient samples. (D) Similar to (C), but for germline enhancer-associated SV breakpoints in *ZNF462* 3′ UTR. For (B), SV-expression association FDRs correct for tissue type (by TCGA or ICGC project), gene-level copy, tumor ploidy, and tumor purity by linear modeling. For (C and D), differential expression p values correct for tissue type (tissue type categories as provided in the same figure), and boxplots represent 5%, 25%, 50%, 75%, and 95%.

Regulatory regions within the gene 3′ UTR can influence the stability of the corresponding mRNA, e.g., via binding by microRNAs or regulatory proteins^29^ (Figure 4A). In addition to the above genomic region window examined in relation to genes (Figure 2A), we assessed germline SV breakpoints falling within the gene 3′ UTR associated with altered mRNA levels (Table S3). At an FDR of 10% or lower (linear model correcting for covariates), seven genes—*METTL21A*, zinc finger protein 100 (*ZNF100*), *HLA-DQB2*, zinc finger protein 462 (*ZNF462*), *KRTAP4-8*, *BTBD19*, and *TMEM105*—had significantly increased expression in relation to 3′ UTR-associated breakpoints spanning various tissues (Figure 4B), where the normal degradation or regulatory processes could conceivably be lost in the affected patients. As examples, *ZNF100* involved 645 patients with a recurrent deletion SV having one breakpoint located in the 3′ UTR (Figure 4C), and *ZNF462* involved 38 patients with a recurrent inversion SVs having both breakpoints located in the 3′ UTR (Figure 4D). In addition to the above analysis involving germline SVs, we also carried out a similar 3′ UTR analysis for the PCAWG somatic SVs (Table S5). Only one gene, *BCL2*, had a significant positive association (FDR of <10%), involving 29 lymphomas and two leukemias, which association involved an already well-known translocation t(14; 18) juxtaposing *BCL2* to the immunoglobulin heavy chain gene enhancer at band 14q32, resulting in gene over-expression and inhibition of apoptosis.^30^

### Expression alterations involving both germline and somatic SV breakpoints

**Figure 5:**
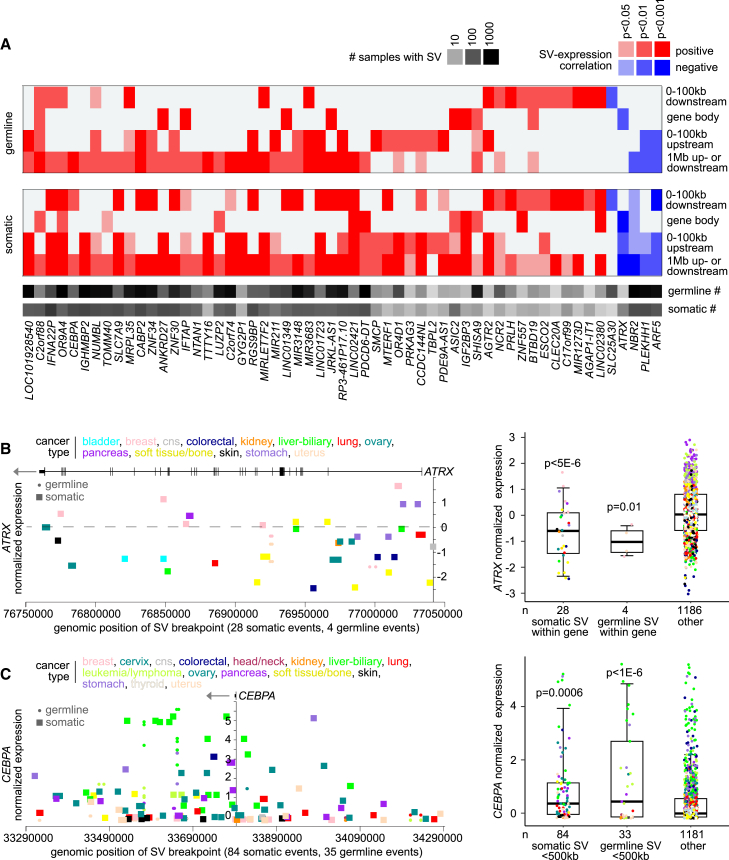
Genes with altered expression recurrently associated with both germline and somatic SV breakpoints (A) Heatmap of significance patterns for 57 genes with both germline and somatic SV breakpoints associated with altered expression (p < 0.01 for each by linear modeling correcting for covariates) in the same direction and for the same genomic region window (100 kb upstream of the gene, 100 kb downstream of the gene, within the gene body, or 1 Mb upstream or downstream of the gene). (B) *ATRX* mRNA levels in tumors corresponding to germline SV breakpoints (circles) and somatic SV breakpoints (squares) located within the gene (left). Boxplot (right) shows *ATRX* expression by patient samples with somatic SV breakpoints within the gene versus samples with germline SV breakpoints within the gene versus other patient samples. (C) Similar to (B), but for germline and somatic SV breakpoints located 1 Mb upstream or downstream of *CEBPA*. For (A), SV-expression association p values correct for tissue type (by TCGA or ICGC project), gene-level copy, tumor ploidy, and tumor purity by linear modeling. For (B and C), differential expression p values versus other group correct for tissue type (tissue type categories as provided in the same figure), and boxplots represent 5%, 25%, 50%, 75%, and 95%.

Both somatic and germline SVs may contribute to the tumor’s molecular profile. In parallel with the above analyses linking germline SV breakpoint patterns with altered expression of nearby genes, we carried out a similar set of analyses for the PCAWG somatic call set (Table S5). Many more genes had significant somatic SV-expression associations than had germline SV-expression associations (even with relatively fewer somatic versus germline SVs represented in the PCAWG datasets). For somatic SV breakpoints in regions 100 kb upstream of the gene, 100 kb downstream of the gene, within the gene body, or 1 Mb upstream or downstream of the gene, the numbers of significant genes at a FDR of less than 10% were 1,288, 1,152, 913, and 1,187, respectively, after correction for tumor tissue of origin, tumor ploidy, tumor purity, and gene-level copy number. Notably, there was little overlap in the significant genes between the germline and somatic SV results sets. A set of 57 genes had both germline and somatic SV breakpoints associated with altered expression (p < 0.01 for each by linear modeling correcting for covariates) in the same direction and for the same genomic region window (Figure 5A).

Genes of particular interest within the set of 57 included two genes with well-established cancer associations by COSMIC^25^: alpha-thalassemia mental retardation X-linked (*ATRX*) and CCAAT enhancer binding protein alpha (*CEBPA*). *ATRX* is one of the most frequently somatically mutated tumor suppressor genes in human cancers, with roles in regulating chromatin state, gene expression, and DNA damage repair.^31^ Both somatic and germline SV breakpoints within *ATRX* were associated with lower gene expression, resulting from gene disruption (Figure 5B). Of the 1,218 PCAWG patients, 28 had tumors harboring somatic SV breakpoints within *ATRX* (spanning multiple cancer types), and an additional four patients (two breast cancer and two uterine cancer) had germline SV breakpoints within *ATRX*, with *ATRX* expression notably lower in tumors from both patient groups (Figure 5B). In contrast, both somatic and germline SV breakpoints spanning the genomic region within 500 kb of the gene start of *CEBPA* were associated with higher gene expression (Figure 5C). Of the 1,218 PCAWG patients, 84 had tumors harboring somatic SV breakpoints for the above genomic region, and an additional 33 patients had germline SV breakpoints, with *CEBPA* expression elevated in tumors from both patient groups, including liver tumors (Figure 5C). Interestingly, *CEBPA* itself is understood to represent a tumor suppressor gene, particularly in leukemia.^32^ However, long non-coding RNA *CEBPA-DT*, a divergent transcript of *CEBPA*, has recently been found to promote liver cancer metastasis through DDR2/β-catenin activation.^33^^,^^34^

### Germline SV-associated expression involving genes essential in cell lines

**Figure 6:**
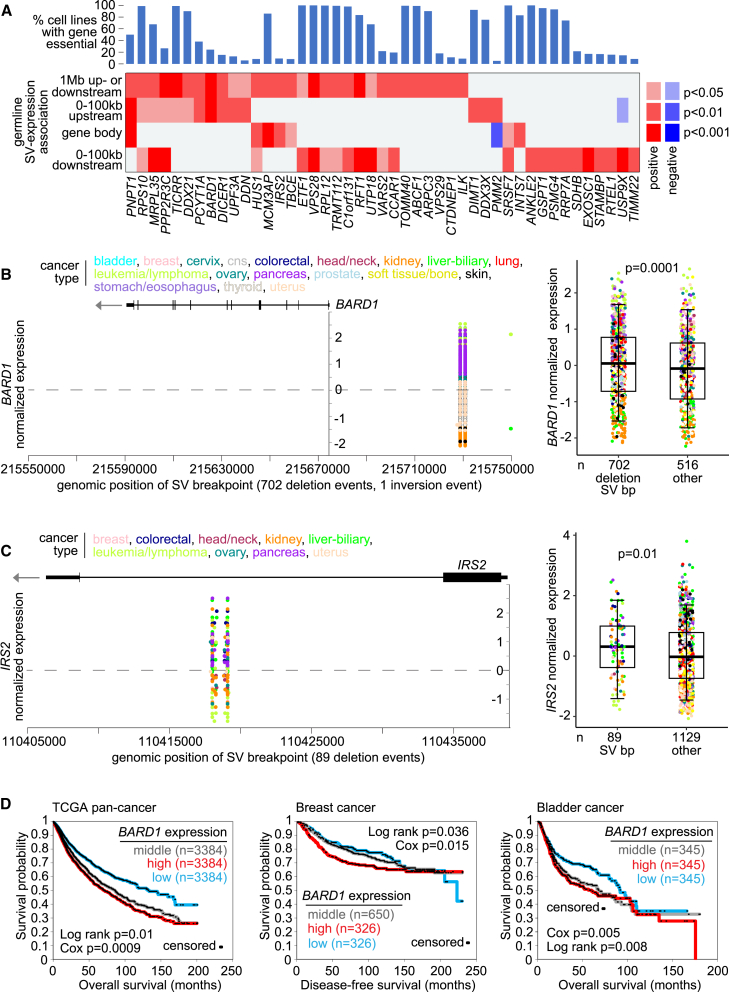
Germline SV breakpoint patterns involving essential genes in cancer cell lines (A) Combining germline SV data with CRISPR knockout screening results from the DepMap project of 1,070 cancer cell lines,^35^^,^^36^ a set of 45 genes had both germline SV breakpoints associated with increased expression (p < 0.01 for any region by linear modeling correcting for covariates) and were found essential (with gene effect score of <−0.75) in more than 5% of cell lines. (B) *BARD1* mRNA levels in tumor samples corresponding to germline SVs located in the genomic region 100 kb upstream of the gene (left). Each SV for a given patient sample has two breakpoints represented. Boxplot (right, representing 5%, 25%, 50%, 75%, and 95%) shows *BARD1* expression by tumor samples with germline SV breakpoint downstream of the gene versus other tumors. p value by linear model correcting for tumor tissue of origin with covariates. (C) Similar to (B), but for germline SV breakpoints located within the *IRS2* gene. (D) Association of *BARD1* expression with worse patient outcome in TCGA pan-cancer (n = 10,152 patients),^37^ breast cancer (n = 1,302),^38^ and bladder cancer (n = 1,035)^24^ cohorts. The p values are by log rank test and by Cox. For the TCGA pan-cancer dataset, tests correct for cancer type (by TCGA project). For breast dataset, survival is capped at 230 months.

To further sift through the germline SV-expression associations for genes of particular interest, we turned to the Cancer Dependency Map (DepMap) CRISPR assays^35^^,^^36^ measuring the essentiality of each gene for each of 1,070 cancer cell lines. A low DepMap-based gene effect score for a given gene in a cell line indicated that the cell line is dependent on the gene for proliferation *in vitro*. We crossed the DepMap results with the set of 580 genes having germline SV breakpoints associated with increased expression (p < 0.01 for any region by linear modeling correcting for covariates, involving at least three patients with SV breakpoints). Of the 580 genes, 45 genes were found essential (with gene effect score of <−0.75) in more than 5% of cell lines (Figure 6A, Table S3). The 45 genes did not represent a statistically significant overlap between the respective results sets, consistent with the notion that most genes involving germline SV-expression association would not pertain directly to cancer, as well as the fact that the CRISPR assays are limited to measuring a specific variable in an artificial model system. Still, the 45 genes represent potential cancer therapeutic targets, including several genes of interest, as explored below. Some genes were essential in more than 90% of cell lines, including genes encoding RNA binding proteins and ribosomal components, while other genes were essential in just a small fraction of cell lines, possibly reflecting genetic variation.

BRCA1 associated RING domain 1 (*BARD1*) has often been thought of as a tumor suppressor gene, but recent studies have caused some to re-evaluate its cancer-related role as being oncogenic instead, with the gene having potential as a therapeutic target for cancer susceptibility and testing.^39^ *BARD1* was essential in 24% of cell lines by DepMap, while a germline SV 100 kb upstream of the gene—involving 702 patients—associated with its increased expression (Figure 6B). Another gene, insulin receptor substrate 2 (*IRS2*), was essential in 9% of cell lines, with an intronic germline SV—involving 89 patients—being associated with increased expression (Figure 6C). *IRS2* is a transforming oncogene that encodes an adaptor protein in the insulin-like growth factor I receptor pathway, with implications for therapeutic targeting.^40^ *DICER1*, encoding the Dicer enzyme that cleaves double-stranded RNA and pre-microRNA into small interfering RNA and microRNA, respectively,^41^ had germline SV breakpoints further away from the gene—up to ∼787 kb upstream or downstream and involving 51 patients—associated with its increased expression (Figure S4A). However, the *DICER1*-associated SV breakpoints did not fall into a tight pattern of location as observed for the other genes of interest in this study. Furthermore, greater *BARD1* expression associated with worse patient outcome across multiple cancer types (Figures 6D and S4B), as observed across the entire TCGA pan-cancer cohort (n = 10,152 patients, correcting for survival differences by cancer type cancer type),^37^ as well as for individual cancer types, including breast cancer (n = 1,302)^38^ and bladder cancer (n = 1,035),^24^ representing other patient cohorts. *BARD1* also trended with worse outcome in pediatric brain tumors (Figure S4C). The association of *BARD1* expression with worse patient outcome, along with the gene being essential in a large percentage of cell lines, would seem to be consistent with the gene having an oncogenic role.

### SVs and associated genes involving patient survival

While most genes with germline SV-associated altered expression would presumably have no role in cancer, we hypothesized that a subset of genes with potential roles might be uncovered through cancer patient survival analyses across molecular datasets from different cohorts. We, therefore, identified the subset of genes with germline SV-associated altered expression for which the germline SV breakpoint patterns were also associated with patient overall survival in the PCAWG cohort. For each of the 22,956 genes in our PCAWG datasets, we associated the germline SV breakpoint pattern with survival (by Cox analysis, correcting for tumor tissue of origin) for each of the SV breakpoint matrices respectively involving regions 100 kb upstream of the gene, 100 kb downstream of the gene, within the gene body, or 1 Mb upstream or downstream of the gene (Table S6). For this analysis, we required a positive association between breakpoint pattern and survival, as the absence of a germline SV should presumably not increase risk of a shorter time to adverse event. Consistent with this assumption, we found many more genes with significant positive associations than negative associations of SV breakpoint with survival, with, for example, 1,381 positive versus 218 negative genes significant (two-sided p < 0.05) for the 1-Mb region (Table S6).

**Figure 7:**
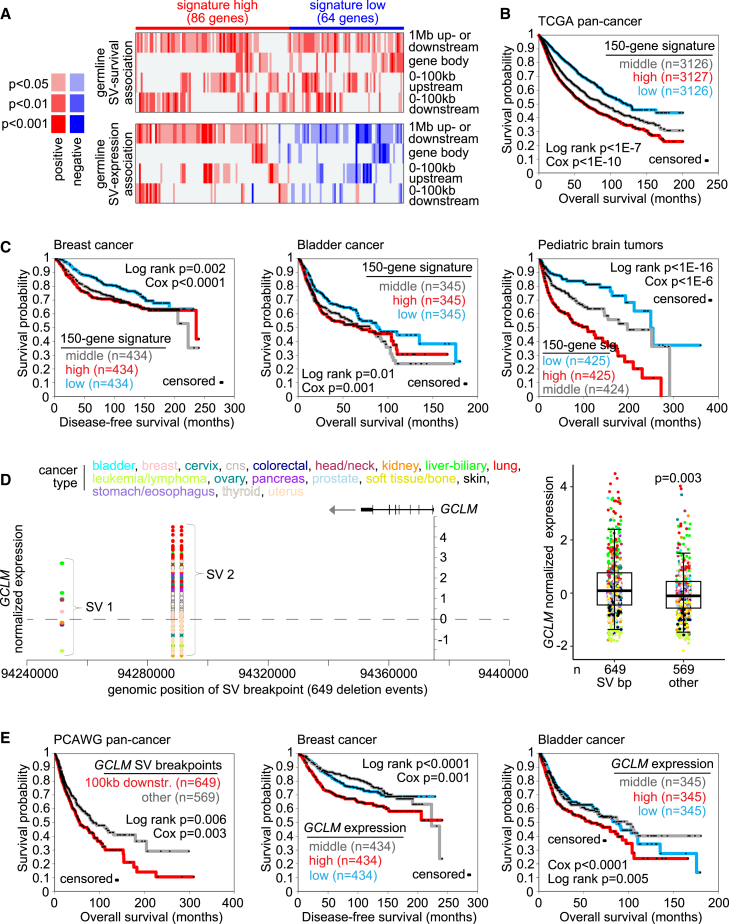
Germline SV breakpoint patterns involving cancer patient survival (A) Combining germline SV data with patient survival data and tumor expression data across the 1,218 PCAWG patients, 150 genes had both a positive association between germline SV breakpoints near the gene and worse overall survival and a positive or negative germline SV-expression association. Genes listed have significant survival association of one-sided p < 0.05 by Cox (corrected for tissue type) for any of the indicated genomic region windows examined, and significant for expression association for any genomic region window with p < 0.05 by linear model correcting for tumor project, gene-level copy, tumor ploidy, and tumor purity. (B) Association of the 150-gene signature from part a with patient survival in TCGA pan-cancer dataset (n = 9,379, no PCAWG tumors),^37^ based on scoring of the tumor expression profiles. The direction of each gene in the 150-gene signature, as applied here to the entire TCGA cohort, is based on the direction of the germline SV-expression association. Survival association p values correct for cancer type. (C) Association of the 150-gene signature from part a with patient survival across multiple cancer types and three separate expression datasets: breast cancer (n = 1,302),^38^ bladder cancer (n = 1,035),^24^ and pediatric brain tumors (n = ,1274).^22^ The p values were corrected by histological type for the pediatric brain dataset. (D) *GCLM* mRNA levels corresponding to germline SVs located in the genomic region 100 kb downstream of the gene (left). Each SV for a given patient sample has two breakpoints represented. Boxplot (right, representing 5%, 25%, 50%, 75%, and 95%) shows *GCLM* expression by tumor samples with germline SV breakpoint downstream of the gene versus other tumors. The p value is by linear model correcting for tumor tissue of origin. (E) Association of *GCLM* germline SV breakpoint patterns with worse patient outcome, and association of *GCLM* expression with worse patient outcome in breast and bladder cancer cohorts. For the SV breakpoints dataset, tests correct for tumor tissue of origin. For (A), Cox p values are one sided; all other Cox p values in the figure are two sided.

Across the PCAWG patients, we identified a top set of 150 genes for which there was both a positive association (for any region examined) between germline SV breakpoints near the gene and worse overall survival (one-sided p < 0.05, Cox correcting for tissue of origin) and a positive or negative association (two-sided p < 0.05 by linear model, any region) between nearby germline SV breakpoints and altered gene expression (Figure 7A). Of the 150 genes, 86 had a positive association between breakpoint pattern and expression, and 64 had a negative association. The above would represent a subset of germline SV-expression associations of potential interest due to the survival associations. However, as we had not used expression associations with survival to derive the 150 genes, we tested whether the 150-gene signature could collectively predict patient outcome in external molecular datasets of cancer gene expression. Taking the direction of the germline SV-expression association as the direction of each gene in the 150-gene signature, we examined the signature association with outcome in the TCGA pan-cancer expression dataset (Figures 7B and S5A) (n = 9379 patients, no PCAWG tumors, and correcting for survival differences by cancer type)^37^ and three additional expression datasets (Figures 7C and S5B): breast cancer (n = 1,302),^38^ bladder cancer (n = 1,035),^24^ and pediatric brain tumors (n = 1,274).^22^ For each external dataset, the 150-gene signature could distinguish higher risk from lower risk patients with statistical significance (Figures 7B and 7C).

One example of a gene in the 150-gene signature (Figure 7A) that was individually associated with patient overall survival was *GCLM* (glutamate-cysteine ligase modifier subunit). Germline SVs with breakpoints 100 kb downstream of *GCLM* involved two distinct recurrent SV deletion events, with breakpoints in 649 patients associated with increased expression (Figure 7D). *GCLM* also showed associations with worse patient survival in terms of both breakpoint patterns and expression (Figures 7E and S5C). A recent study provided experimental support for *GCLM* as a tumor promoter in bladder cancer.^42^ In parallel with the above analyses linking germline SV breakpoint patterns with patient survival (Figure 7A), we carried out a similar set of analyses for the PCAWG somatic call set, from which 595 genes had somatic SV breakpoints both positively associated with worse overall survival and positively or negatively associated with altered expression (Figure S6A). The 595 genes could also predict patient survival in external patient cohorts based on gene expression (Figures S6B–S6E). Unlike the 150 germline SV-associated genes, however, the 595 somatic SV-associated genes could not predict survival in pediatric brain tumors (Figure S6C), which related diseases might be expected to have a stronger germline component than that of adult cancers. In addition, only one gene, G protein-coupled receptor 132, was in both the germline SV-related 150-gene set and the somatic SV-related 595-gene set (Table S6).

## Discussion

Germline structural variation is a major source of gene expression differences in humans.^10^ Here, we assembled a catalog of gene-level germline SV-expression associations in human tumors, in contrast to similar types of catalogs previously based on somatic SVs.^14^^,^^15^^,^^17^^,^^21^^,^^22^^,^^24^ Our study of germline SVs represented an opportunity to characterize a class of normal variation across individuals that extends beyond SNVs. Most of the significant germline SV-associated genes we identified would not necessarily have specific roles in cancer, but, for some genes, the expression variation might have some other phenotype. At the same time, several genes impacted by germline SVs could conceivably contribute to cancer, e.g., through these genes having an established cancer association or an association with patient survival. Cancer-relevant genes involving germline SVs include *ATRX*, *CEBPA*, *BRD1*, *IRS2*, and *GCLM*. For germline SVs, we observed here that the absolute expression differences between SV-associated and other patients may be relatively small, but still statistically significant, in contrast with somatic SVs that often involve dramatic expression changes. Somatic SVs represent random mutations with often catastrophic consequences, whereas germline SVs would represent a normal class of genetic variation passed down through generations.

The SVs arising in our study might also be explored elsewhere for any possible cancer risk associations by GWAS and similar studies involving both cases and controls. The subset of genes with cancer-relevant associations arising in our present study would represent strong candidates for further investigation. Historically, GWAS and similar studies have primarily focused on SNVs, not SVs.^43^ As WGS is applied more in future studies, germline SVs should receive more attention. The results of this present study can help guide future genomic case-control studies, which studies could benefit from increased statistical power when focusing on a select set of germline SVs from our study versus examining all variants.^44^^,^^45^ Conversely, any significant germline SVs that may arise in case-control association studies can be crossed with our results to determine any related expression changes that might plausibly explain the association. In our previous studies of somatic SVs, we first identified many associations later established in subsequent studies, such as those involving *TERT*^14^^,^^46^^,^^47^ and programmed cell death 1/programmed cell death ligand 1 genes.^14^^,^^48^ In the clinical setting, any established cancer risk variants could potentially aid in genetic testing, with a subset of variants found here to also involve essential genes with targeting implications. Many germline SV-expression associations identified in the PCAWG cohort could also be relevant in other cellular or phenotypic contexts outside of cancer.

While some SVs may involve gene duplication, deletion, or disruption, others involve *cis*-regulatory alterations. Like the eQTL approach, our approach to SV-expression associations does not assume the mechanism for altered gene *cis*-regulation. However, specific mechanisms would be discoverable by mining our associations to identify patterns of enrichment, e.g., as we did here regarding enhancer duplication events. For somatic SVs, mechanisms of SV-mediated altered *cis*-regulation are diverse and would include disruption of topologically associated domains, enhancer hijacking, and altered DNA methylation.^17^ In contrast, germline SVs would have fewer possibilities for altering the regulatory landscape, as germline SVs involve very few translocations and are smaller in size than somatic SVs. Most of the germline SV-expression associations we identified did not have a likely mechanism assigned, representing an avenue for future work. Outside information can shed light on possible mechanisms, although germline SV-expression associations show but modest enrichment at regulatory elements and remain difficult to predict from current annotations.^7^ Still, as additional annotations or information emerge, our results, which represent a resource, can be re-examined in this light.

### Limitations of the study

While cancer-relevant genes would be included in our results, our present study cannot definitively link a specific germline SV to increased cancer risk. Any disease or phenotypic relevance regarding our germline SV-expression associations remains to be elucidated. Future case-control studies focusing on our study’s top SVs of interest could help to establish any hypothesized cancer risk associations. Whether the observed small expression changes in cancer-relevant genes over the course of a patient’s life could contribute to cancer remains a question for further study. As cancer genomics datasets with combined WGS and expression data and much greater patient numbers become available, a more refined catalog of SV-expression associations can be assembled with the benefit of increased statistical power.^49^ Germline SV-expression associations involving specific cancer types could also be explored using greater patient numbers and more extensive outcomes data.

## STAR★Methods

### Key resources table

**Table:** 

REAGENT or RESOURCE	SOURCE	IDENTIFIER
**Deposited data**		

PCAWG WGS and expression datasets	International Cancer Genome Consortium (ICGC) Data Portal Release 28	https://dcc.icgc.org/releases/release_28
Cancer Dependency Map (DepMap) CRISPR assays	Chronos algorithm-generated dataset	https://github.com/broadinstitute/chronos
The Cancer Genome Atlas (TCGA) transcriptomic datasets	Broad Institute	https://gdac.broadinstitute.org/
breast cancer transcriptomic datasets	Gene Expression Omnibus (GEO)	GSE12093, GSE2034, GSE69031, GSE7390, GSE5327, GSE2603, GSE11121, GSE6532
bladder cancer transcriptomic datasets	Gene Expression Omnibus (GEO) and Broad Institute (TCGA)	GSE13507, GSE31684, GSE32894, GSE48075, GSE48276, TCGA BLCA
CBTN transcriptomic data	Kids First Data Resource Portal and Cavatica	https://portal.kidsfirstdrc.org https://cavatica.squarespace.com/

**Software and algorithms**		

SVExpress (v1.0)	Baylor College of Medicine	https://github.com/chadcreighton/SVExpress https://doi.org/10.5281/zenodo.10592237

### Resource availability

#### Lead contact

Further information and requests for resources and reagents should be directed to and will be fulfilled by the lead contact, Chad J. Creighton (creighto@bcm.edu).

#### Materials availability

This study did not generate new unique reagents.

#### Data and code availability

This paper analyzes existing, publicly available data. Details on accessing the datasets are listed in the key resources table. This paper does not report original code. No custom computer code was used for data collection, which was performed using open-source software. Additional processing involved in-house scripts that are available upon request. All analyses used previously published software or methods.

Any additional information required to reanalyze the data reported in this paper is available from the lead contact upon request.

### Experimental model and study participant details

Regarding human subjects, cancer molecular profiling data were generated through informed consent as part of previously published studies and analyzed in accordance with each original study’s data use guidelines and restrictions. The results here are based on data from the Cancer Genome Atlas (TCGA) Research Network and the International Cancer Research Consortium (ICGC).

### Method details

#### Patient datasets

Datasets of germline and somatic structural variants (SVs), RNA expression, and copy number were generated as part of the Pan-Cancer Analysis of Whole Genomes (PCAWG) project^6^. Germline and somatic SV and gene expression data were compiled and harmonized by the PCAWG initiative from 29 previous studies (Table S1). Of the 2658 PCAWG donors, 1220 had RNA data,^14^ of which 1218 had corresponding WGS-based germline somatic SV calls by both Delly^50^ and Snowman (SvABA)^51^ algorithms (Table S1, two TCGA cases of the 1220 not having germline SV calls by Snowman algorithm). Tumors profiled spanned a range of cancer types (bladder, sarcoma, breast, liver-biliary, cervix, leukemia, colorectal, lymphoma, prostate, esophagus, stomach, central nervous system, head/neck, kidney, lung, skin, ovary, pancreas, thyroid, uterus), as detailed in Table S1. In the minority of patients with multiple tumors profiled, we selected one tumor to represent the patient.

Germline SV variant call files, generated by the PCAWG consortium, were downloaded from the Cancer Genome Collaboratory (Collab, ICGC cases) and the Protected Data Cloud (PDC, TCGA cases). We compiled germline SV calls in the final analysis set after filtering for calls by both of two algorithms. Germline SV calls from Snowman (Broad pipeline) and Delly (DKFZ pipeline) were pairwise joined based on SV position, allowing 200 bp slop at the breakpoints. For this merged germline SV call set, we used the Snowman SV genomic coordinates throughout the study. For somatic SVs, calls were made by three different data centers using different algorithms; calls made by at least two out of four algorithms were used in the downstream analyses, along with additional filtering criteria as described by the PCAWG consortium.^6^ Somatic SVs were defined by comparison between the tumor and matched normal. For copy number, the calls made by the Sanger group using the Ascat NGS algorithm^6^ with default parameters, which data are available at the ICGC Data Portal (https://dcc.icgc.org/releases/release_28). Tumor ploidy and purity (tumor cell fraction) values were provided by PCAWG6. For RNA-seq data, alignments by both STAR (version 2.4.0i,2-pass) and TopHat2 (version 2.0.12) were used to generate a combined set of expression calls.^52^ Quantification on a per-transcript level was performed with Kallisto (v.0.42.1), and Gencode v.19 was used as the gene annotation reference. PCAWG consortium performed quantification of consensus expression by taking the average expression based on STAR and TopHat2 alignments. Gene counts were normalized by adjusting the counts to Fragments Per Kilobase per Million mapped fragments (FPKM) as well as FPKM with upper quartile normalization (FPKM-UQ) in which the total read counts in the FPKM definition has been replaced by the upper quartile of the read count distribution multiplied by the total number of protein-coding genes. Alignment parameters and other methodology details are provided at ref. 53. FPKM-UQ values were used in the present study (dataset available at https://www.synapse.org/#!Synapse:syn5553991).

#### Integrative analyses between SVs and expression

Using SVExpress,^23^ we defined genes with altered RNA expression associated with nearby SV breakpoints. These analyses were carried out separately for both germline and somatic SV call sets. Relative to each gene, genomic region windows considered included the within-gene regions and within 100kb upstream or 100kb downstream of the gene, as well as gene 3′UTR. For the above regions, SVExpress constructed a gene-to-sample matrix with entries as 1 if a breakpoint occurs in the specified region with respect to the given gene in the given sample, and 0 if otherwise. We also used SVExpress to examine a 1Mb region surrounding each gene (1Mb upstream or downstream of the gene start), using the “relative distance metric” option,^17^ whereby breakpoints close to the gene will have more numeric weight in identifying SV-expression associations, while breakpoints further away but within 1Mb can have some influence. Gene-level SV-expression association analyses included 22956 uniquely identified genes (with Entrez gene identifier). Using the geneXsample SV breakpoint matrix, SVExpress assessed the correlation between expression of the gene and the presence of an SV breakpoint using a linear regression model (with log-transformed expression values). Linear regression models separately evaluated significant associations without any correction for covariates, or when correcting for tumor tissue type, or when correcting for tissue type, gene-level copy number, tumor ploidy, and tumor purity. Genes significant by the third model—correcting for tissue type, gene-level copy number, tumor ploidy, and tumor purity—were carried forward in the downstream analyses.

By SVExpress, a gene shows significant SV-expression associations if the expression and SV breakpoint patterns line up non-randomly with respect to each other across all samples analyzed, after correction for covariates. By design,^17^ our integrative analytical approach does not assume the specific mechanism of altered expression, treating SV breakpoints representing different classes (duplications, deletions, inversions, and translocations) and insert sizes the same.^21^ For the 1 Mb region, if multiple breakpoints occur near the gene, the breakpoint closest to the gene start is used in the breakpoint matrix. For the analyses involving within-gene, 100kb upstream, and 100kb downstream gene regions, we only considered genes with at least three tumors associated with an SV within the given region when estimating False Discovery Rate.^45^ All genomic coordinates were based on the hg19 human reference genome. To identify potential enhancer duplication events involving gene-level SV-expression associations, we used the enhancer annotations provided by Kumar et al.^28^ Gene 3′UTR positions were obtained from Ensembl biomart.^53^

Our analytical approach to associate SVs with gene expression was gene-centric rather than variant-centric. This aspect is in contrast with eQTL-based approaches focusing on variant-level associations.^44^ A gene-centric approach simplifies the number of tests performed. Our approach also allows for different variants within a region to collectively contribute to altered expression, which lends itself well to the study of somatic SVs in particular, as somatic SVs are usually not recurrent but can have breakpoints covering a sizable genomic region in relation to genes.^16^ In contrast, germline SVs tend to be highly recurrent and common, and in some cases, only certain germline SVs and not others within a given region would be associated with altered expression of nearby genes. Furthermore, our approach would identify associations that cut across tumors of different tissues of origin, whereas eQTL associations are typically within a specific tissue or cell type.^7^^,^^10^ Our approach incorporates tissue of origin as a covariate, allowing any significant genes to have different baseline expression levels between tissues. We observed significant overlap between our results and previous results from the eQTL approach obtained using GTEx.^7^ Differences between the respective results sets could involve tumors representing highly aberrant cellular systems, where cell- or tissue-specific programs may be lost, e.g., due to loss of differentiation.

#### Survival analyses

We identified gene-level molecular correlates of patient survival associated with nearby germline SV breakpoints in the PCAWG cohort of 1218 patients. For associating nearby SV breakpoints with patient outcome, we utilized the geneXsample relative distance breakpoint matrix, generated by SVExpress, for a given genomic region in relation to the gene (within the gene boundary, 100kb upstream of the gene, 100kb downstream of the gene, or within 1Mb of the gene start, with relative distances involving the 1Mb region being weighted using the “relative distance metric” option^17^). For each gene, we used a stratified Cox (correcting for cancer type) to associate patient overall survival with the germline SV breakpoint patterns for that gene. We also associated mRNA expression of the gene with overall survival using stratified Cox (corrected for cancer type, using as.factor in R). We compared the set of genes significant for the SV breakpoint survival association analysis with the set of genes with SV-mRNA associations. When overlapping different result sets, we used more relaxed p value cutoffs to limit false negatives, where the overlapping genes were yielded significant expression-based survival associations in multiple external datasets. In addition to the associations of germline SV breakpoint patterns with patient outcome, we carried out an analogous set of analyses for the somatic SV breakpoint patterns.

We also examined genes and gene sets of interest in public cancer transcriptomic datasets for associations between expression and patient outcome. To analyze breast cancer patient survival, we used a compendium expression dataset involving 1302 patients and nine separate datasets assembled previously.^38^ To analyze bladder cancer patient survival, we used a compendium expression dataset involving 1035 patients and five separate datasets assembled previously.^24^ We used RNA-seq data from the Children’s Brain Tumor Network (CBTN)23 to analyze pediatric brain tumor patient survival. For CBTN, we separately generated results for both the initial RNA dataset^22^ and the more recent “X01” dataset; then, we combined the respective results in associating gene signatures with patient outcome. The TCGA pan-cancer RNA-seq dataset, representing 32 major cancer types and 10224 tumors, was assembled as previously described.^37^ Patients represented in both TCGA and PCAWG datasets were not included in the TCGA pan-cancer survival analyses, leaving 9379 patients with combined RNA expression and survival data. For analyses involving the TCGA pan-cancer and breast compendium datasets, patient survival was capped at 200 months and 240 months, respectively, except where indicated. For the CBTN dataset, we included only one tumor per patient in the survival analyses.

Given a gene signature (e.g., the 150-gene signature of Figure 7A), we scored patient profiles in the external expression dataset using our previously described “t score” metric.^54^^,^^55^ This t score represents the two-sided t statistic when comparing, within each external differential expression profile, the average of the signature high genes with the average of the signature low genes. For example, the t score for a given sample profile is high when the genes high and low in the signature are respectively high and low on average in the external sample profile. We assessed the association of the expression of individual genes or the gene signature score with patient outcome using Cox and log rank (dividing the cases according to low, high, or intermediate signature scoring). In addition, for analyses utilizing the TCGA pan-cancer dataset, stratified Cox models or stratified log rank tests were used to evaluate survival association when correcting for tumor type.

### Quantification and statistical analysis

All p values were two-sided unless otherwise specified. We relied on a stricter FDR cutoff for defining top genes when carrying out gene-level global molecular associations for a single analysis (e.g., gene-level SV-mRNA associations). When overlapping different top-gene results sets (e.g., gene-level SV-expression associations involving enhancer duplication or gene-level SV-expression associations involving both germline and somatic SV analyses), we used a more relaxed p value cutoff to limit false negatives, helping us identify significant overlap patterns. Visualization using heat maps was performed using JavaTreeview (version 1.1.6r4)^56^ and matrix2png (version 1.2.1).^57^ Boxplots represent 5% (lower whisker), 25% (lower box), 50% (median), 75% (upper box), and 95% (upper whisker). Figures represent biological and not technical replicates.

## Supplemental information

Document S1. Figures S1–S6

Table S1. Patient sample annotation table, related to Figure 1 Provided as an Excel file. By patient, sample annotation, with corresponding molecular data and comparisons for genes of interest, is provided for the PCAWG cohort of 1218 patients with combined data by WGS (both tumor and blood normal) and RNA-seq (tumor only). The numbers of SVs per cytoband region for each patient are provided in a separate tab, involving 811 cytoband regions (not including chromosomes X and Y).

Table S2. Germline SVs for genes and samples with recurrently altered expression, related to Figure 2 Provided as an Excel file. Germline SVs and associated samples and genes, for which the genes had a significant association of altered expression with SV breakpoints (linear model, correcting for tissue type by TCGA or ICGC project, gene-level copy, tumor ploidy, and tumor purity) and for which the tumor sample had differential expression in the same direction as the association (greater or less than the median across samples). Germline SV calls of the PCAWG samples were based on detection by both Delly and Snowman algorithms. Corresponding patient identifiers are not provided. For each SV-gene-sample entry, the SV coordinates, gene-level statistics, and relative expression in the sample are indicated. Results are provided for the genomic region windows 100 kb upstream of the gene, 100 kb downstream of the gene, and within the gene. Also noted are any SVs found in one of the four patients with very high numbers of SVs and not in any other patients and not found in DGV, representing only 0.3% of the SV events in this Excel file.

Table S3. Gene-level associations between expression and nearby germline SV breakpoint, related to Figure 2 Provided as an Excel file. Complete set of gene-level associations between mRNA expression and nearby germline SV breakpoint in the PCAWG cohort, according to region examined (e.g., 100 kb upstream, 100 kb downstream, within the gene body, 1 Mb upstream or downstream, gene 3′ UTR, and 2 kb upstream). Results provided using linear regression models evaluated significant associations without any correction for covariates or when correcting for tumor tissue type or for tissue type, gene-level copy, tumor ploidy, and tumor purity. Also provided for each gene is the percentage of the 1070 cell lines in DepMap for which the gene is essential (gene score<−0.75).

Table S4. Germline SV breakpoint patterns for selected genes, related to Figure 2 Provided as an Excel file. Germline SV breakpoint patterns, with associated expression, provided for genes highlighted in the study, including *ATRX*, *CEBPA*, *CRYBB2P1*, *GCLM*, *HLA-DQB2*, *NAGK*, *NUTM2B*, *ZNF100*, *ZNF462*, *BARD1*, *IRS2*. Corresponding patient identifiers are not provided.

Table S5. Gene-level associations between expression and nearby somatic SV breakpoint, related to Figure 5 Provided as an Excel file. Complete set of gene-level associations between mRNA expression and nearby somatic SV breakpoint in the PCAWG cohort, according to region examined (e.g., 100 kb upstream, 100 kb downstream, within the gene body, 1 Mb upstream or downstream, gene 3′ UTR, and 2 kb upstream). Results provided using linear regression models evaluated significant associations without any correction for covariates or when correcting for tumor tissue type or for tissue type, gene-level copy, tumor ploidy, and tumor purity.

Table S6. Gene-level correlations with patient overall survival, related to Figure 7 Provided as an Excel file. By stratified Cox, correcting for cancer type, associations with both nearby germline SV breakpoints and patient overall survival are provided, according to region examined (e.g., 100 kb upstream, 100 kb downstream, within the gene body, 1 Mb upstream or downstream), as well as associations with somatic SV breakpoints and survival. For SV breakpoints, positive Cox beta indicates that nearby breakpoints are associated with worse outcome; negative Cox beta, associated with better outcome. Also provided are gene expression associations with survival for the PCAWG cohort. For expression, positive Cox beta indicates that higher expression is associated with worse outcome; negative Cox beta, associated with better outcome. The 150 genes from Figure 7 are indicated, for which was found both a positive association between germline SV breakpoints near the gene and worse overall survival and a positive or negative association between nearby germline SV breakpoints and altered gene expression.

Document S2. Article plus supplemental information
