# Supplementary material for: Germline structural variation globally impacts the cancer transcriptome including disease-relevant genes
Source: Cell Rep Med. 2024 Mar 4;5(3):101446. doi: 10.1016/j.xcrm.2024.101446 (PMC10983041; doi:10.1016/j.xcrm.2024.101446)
Supplement: Document S1. Figures S1–S6 [file mmc1.pdf]

Cell Reports Medicine, Volume *5*

## Supplemental information

# Germline structural variation globally impacts the cancer transcriptome including disease-relevant genes

**Fengju Chen, Yiqun Zhang, Fritz J. Sedlazeck, and Chad J. Creighton**

Supplementary Figures

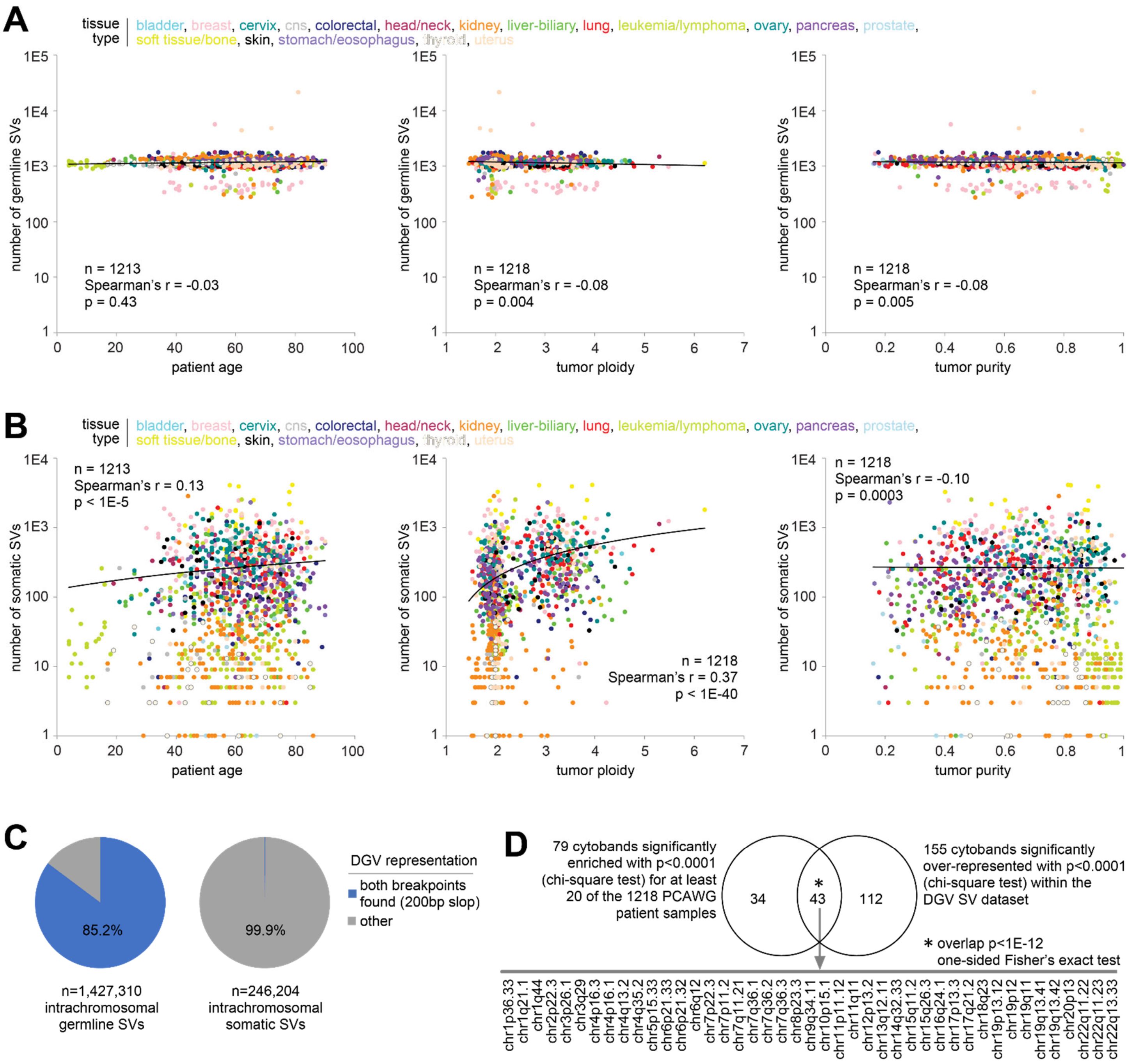

**Figure S1. Additional information regarding germline structural variation patterns in the PCAWG patient cohort. (A)** Across the PCAWG samples with combined RNA (tumor sample) and WGS (tumor sample+blood normal), associations between the total numbers of germline SVs detected in the blood normal of the patient and patient age (left), tumor ploidy (middle), and tumor purity (right). Correlation r-values and p-values by Spearman's. **(B)** Similar to part A, but for the total numbers of somatic SVs detected in the blood normal of the patient. **(C)** For the 1,427,310 intrachromosomal germline SVs and the 246,204 intrachromosomal somatic SVs identified for the 1218 PCAWG patient samples, the fraction of SVs represented in the Database of Genomic Variants (DGV, http://dgv.tcag.ca/dgv/app/home)[1]. DGV database consists of 625,914 germline SVs (minimum size>10, 2020 hg19 version). The DGV SVs were pairwise joined to the PCAWG SVs based on SV position at both breakpoints, allowing 200 bp of slop at the breakpoints. Of the PCAWG germline SVs, 85% were represented in DGV; 55% of PCAWG germline SVs were represented in gnomAD[2] and 56% in the 1000 Genomes Project[3]. **(D)** Cytoband-level enrichment patterns in PCAWG share significant overlap with cytoband-level enrichment patterns in DGV database. Figure 1E assessed cytoband-level enrichment of germline SVs in the PCAWG dataset, with a top set of 79 cytoband regions identified as significant (p<0.0001 by chi-square test) for at least 20 patients. We also evaluated cytoband-level enrichment patterns in the DGV germline SV collection. Of the 79 enriched cytobands from Figure 1E, 43 were also highly enriched in DGV (p<0.0001 by chi-square test), this overlap being highly statistically significant (p<1E-12, one-sided Fisher's exact test, chance expected overlap of ~15). Related to Figure 1.

.

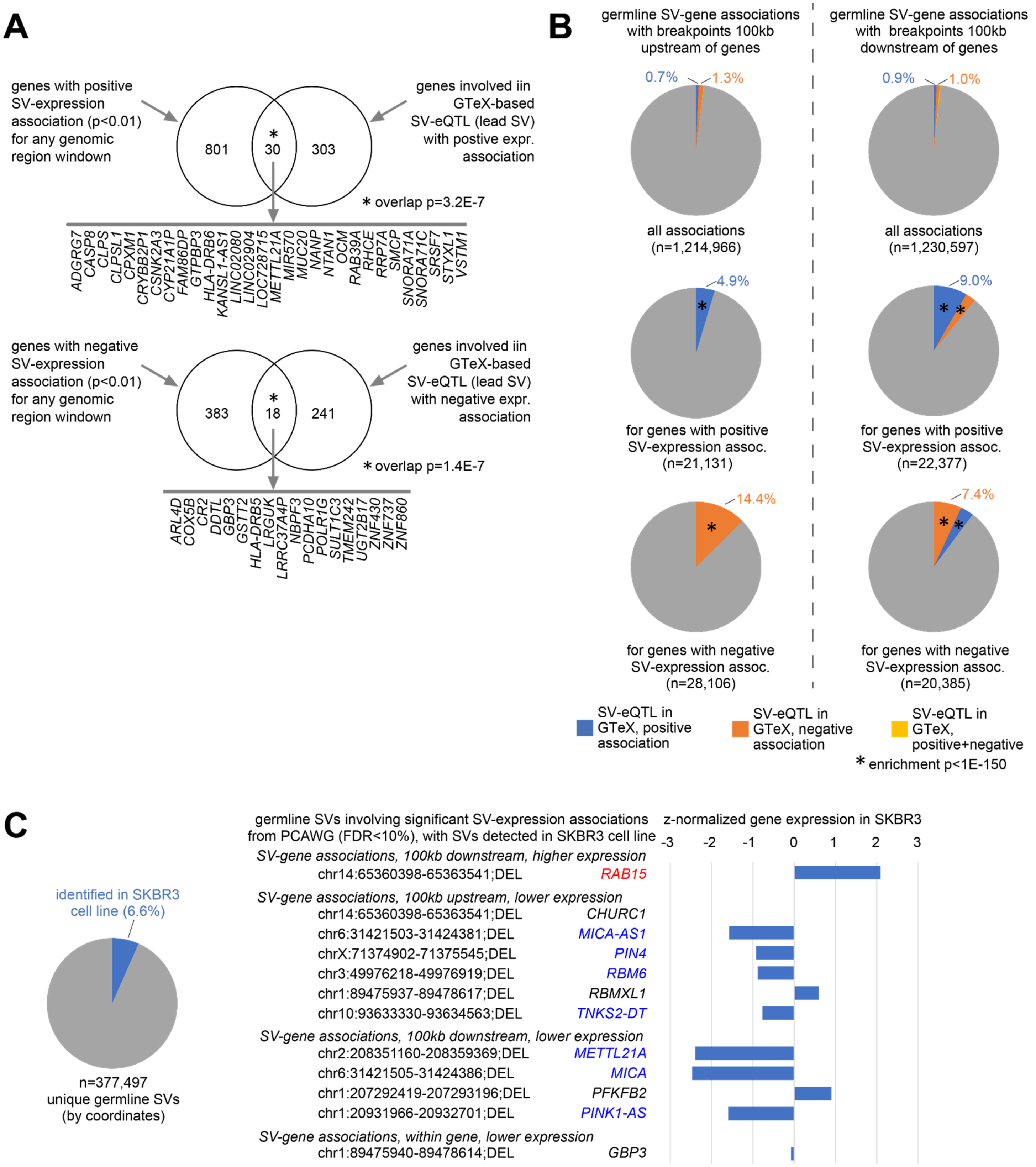
A
genes with positive SV-expression association (p<0.01) for any genomic region windown
genes involved iin GTeX-based SV-eQTL (lead SV) with postive expr. association
801
*
30
303
* overlap p=3.2E-7
ADGRG7 CASP8 CLPS CLPSL1 CPXM1 CRYBB2P1 CSNK2A3 CYP21A1P FAM86DP GTPBP3 HLA-DRB6 KANSL1-AS1 LINC02080 LINC02904 LOC728715 METTL21A MIR570 MUC20 NANP NTAN1 OCM RAB39A RHCE RRP7A SMCP SNORA71A SNORA71C SRSF7 STYXL1 VSTM1
genes with negative SV-expression association (p<0.01) for any genomic region windown
genes involved iin GTeX-based SV-eQTL (lead SV) with negative expr. association
383
*
18
241
* overlap p=1.4E-7
ARL4D COX5B CR2 DDTL GBP3 GSTT2 HLA-DRB5 LRGUK LRRC37A4P NBPF3 PCDHA10 POLR1G SULT1C3 TMEM242 UGT2B17 ZNF430 ZNF737 ZNF860
B
germline SV-gene associations with breakpoints 100kb upstream of genes
germline SV-gene associations with breakpoints 100kb downstream of genes
0.7%
1.3%
0.9%
1.0%
all associations (n=1,214,966)
all associations (n=1,230,597)
4.9%
9.0%
for genes with positive SV-expression assoc. (n=21,131)
for genes with positive SV-expression assoc. (n=22,377)
14.4%
7.4%
for genes with negative SV-expression assoc. (n=28,106)
for genes with negative SV-expression assoc. (n=20,385)
SV-eQTL in GTeX, positive association
SV-eQTL in GTeX, negative association
SV-eQTL in GTeX, positive+negative
* enrichment p<1E-150
C
identified in SKBR3 cell line (6.6%)
n=377,497 unique germline SVs (by coordinates)
germline SVs involving significant SV-expression associations from PCAWG (FDR<10%), with SVs detected in SKBR3 cell line
z-normalized gene expression in SKBR3
-3 -2 -1 0 1 2 3
SV-gene associations, 100kb downstream, higher expression
chr14:65360398-65363541;DEL RAB15
SV-gene associations, 100kb upstream, lower expression
chr14:65360398-65363541;DEL CHURC1
chr6:31421503-31424381;DEL MICA-AS1
chrX:71374902-71375545;DEL PIN4
chr3:49976218-49976919;DEL RBM6
chr1:89475937-89478617;DEL RBMXL1
chr10:93633330-93634563;DEL TNKS2-DT
SV-gene associations, 100kb downstream, lower expression
chr2:208351160-208359369;DEL METTL21A
chr6:31421505-31424386;DEL MICA
chr1:207292419-207293196;DEL PFKFB2
chr1:20931966-20932701;DEL PINK1-AS
SV-gene associations, within gene, lower expression
chr1:89475940-89478614;DEL GBP3

**Figure S2. PCAWG-based germline SV-expression associations share significant patterns of overlap with results of outside datasets.** **(A)** A previous study by Scott et al.[4] mapped 61,668 SVs in 613 individuals from the GTEx project and measured their effects on gene expression. Each SV-eQTL in the Scott study involves an SV in a specific tissue type with altered gene expression (higher or lower) for that tissue, these SV-eQTLs involving 1122 germline SVs (minimum length>=10) in total. Considering only the 7960 SV-eQTL associations with an SV as the lead variant (versus an SNV or indel), the Scott SVs were pairwise joined to the PCAWG SVs based on SV position at both breakpoints, allowing 200 bp of slop at the breakpoints. When considering all SV-gene associations, where the SV breakpoint falls either 100kb upstream of the gene (left) or 100kb downstream of the gene (right), pie charts represent the fractions of SV-gene associations involving a GTEx-based SV-eQTL (corresponding to both SV and gene), for both positive and negative SV-eQTLs. As indicated, percentages of PCAWG germline SV-gene associations involving Scott SV-eQTLs were also tabulated for the subset of SV-gene associations with a significant expression association ($p<0.01$ by linear modeling, with tissue type+copy+ploidy correction). Enrichment p-values by chi-square test. **(B)** Venn diagrams representing the significance of overlap, at the gene level, between PCAWG-based and GTEx-based results. From PCAWG, the set of genes with a significant SV-expression association ($p<0.01$, with covariates) for any of the genomic region windows considered (100kb upstream, 100kb downstream, within the gene, 1Mb upstream or downstream) is considered. From GTEx, the set of genes with an SV-eQTL association (with SV as the lead variant) for at least one GTEx tissue is considered. Top diagram is for positively associated genes in both results sets; bottom diagram, for negatively associated genes. Significance of overlap by one-sided Fisher's exact test. **(C)** We referred to a dataset of structural variants previously identified in the SKBR3 breast cancer cell line[5]. Of the 377,497 unique germline SVs identified in the PCAWG dataset (by coordinates, though for different SVs in different samples, the specific coordinates may differ slightly in this tabulation), about 6.6% were also found in SKBR3 cell line (left). Using gene expression data from the Cancer Cell Line encyclopedia (CCLE), we tested whether the SV-gene associations found in the PCAWG dataset with FDR<10% (for regions 0-100kb upstream, 0-100kb downstream, and within the gene) might trend in the same direction in SKBR3 (right). The SVs identified for SKBR3 included 12 involving a PCAWG germline SV-expression association. One of these associations involved increased expression (RAB15), and the other 11 involved decreased expression. SKBR3 had higher expression for RAB15 (expression normalized to other cell lines in the CCLE dataset) and notably lower expression for 7 of the 11 other genes (highlighted using blue text). When using a paired t-test to compare the change of direction of the 12 genes against a null hypothesis of no change (weighting the values according to the anticipated direction of change), the significance p-value is 0.015. Related to Figure 2.

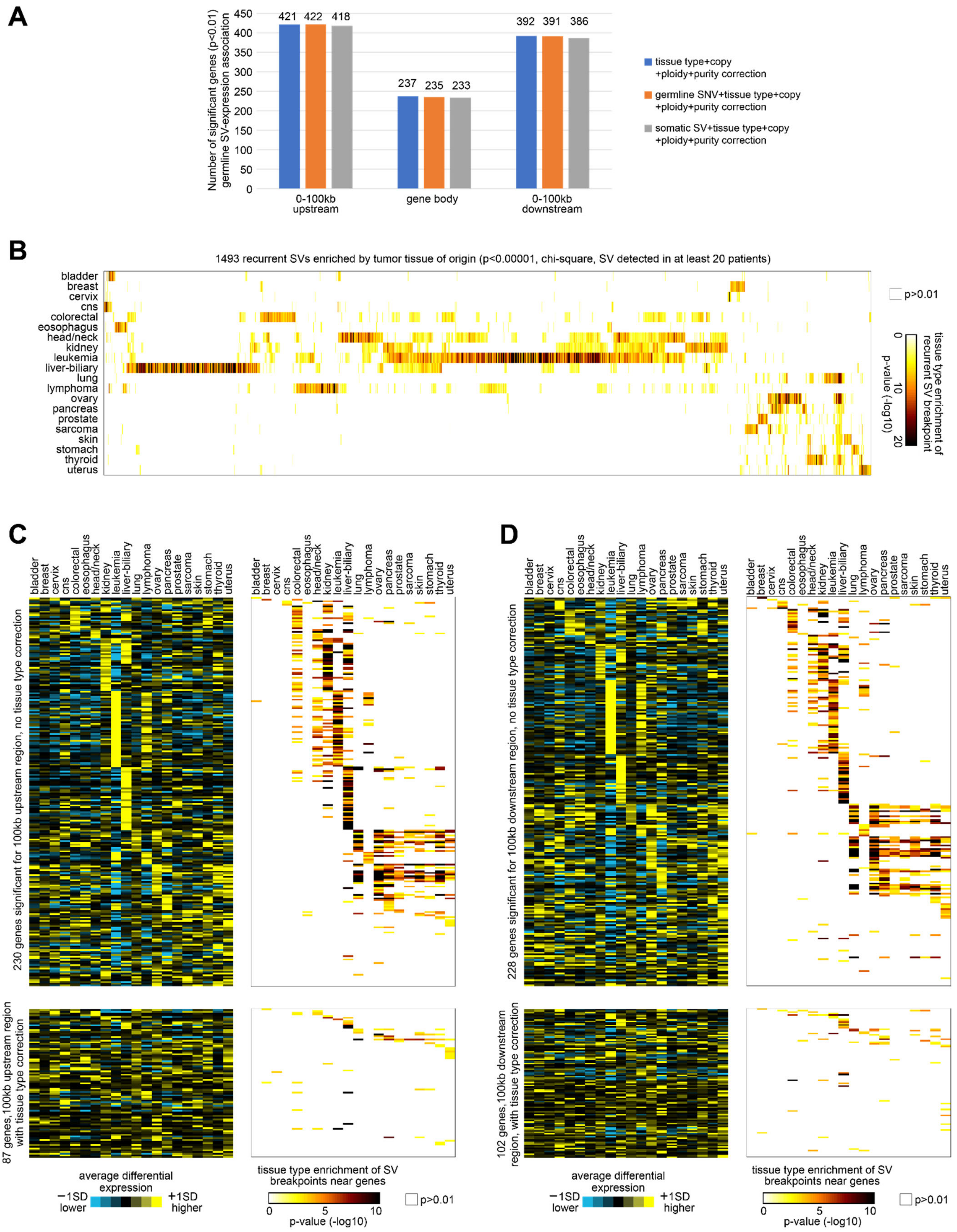
A
Number of significant genes (p<0.01) germline SV-expression association
421 422 418
237 235 233
392 391 386
0-100kb upstream
gene body
0-100kb downstream
tissue type+copy +ploidy+purity correction
germline SNV+tissue type+copy +ploidy+purity correction
somatic SV+tissue type+copy +ploidy+purity correction
B
1493 recurrent SVs enriched by tumor tissue of origin (p<0.00001, chi-square, SV detected in at least 20 patients)
bladder
breast
cervix
cns
colorectal
eosophagus
head/neck
kidney
leukemia
liver-biliary
lung
lymphoma
ovary
pancreas
prostate
sarcoma
skin
stomach
thyroid
uterus
p>0.01
tissue type enrichment of recurrent SV breakpoint
p-value (-log10)
C
230 genes significant for 100kb upstream region, no tissue type correction
87 genes,100kb upstream region with tissue type correction
average differential expression
−1SD lower
+1SD higher
tissue type enrichment of SV breakpoints near genes
p-value (-log10)
p>0.01
D
228 genes significant for 100kb downstream region, no tissue type correction
102 genes,100kb downstream region, with tissue type correction
average differential expression
−1SD lower
+1SD higher
tissue type enrichment of SV breakpoints near genes
p-value (-log10)
p>0.01

**Figure S3. Germline SV-expression associations specific to tumor tissue of origin. (A)** For the set of 1175 genes significant for SV-expression association (p<0.01, linear model correcting for tumor tissue type, gene-level copy, tumor ploidy, and tumor purity) for any of the indicated genomic region windows in relation to genes (100kb upstream of the gene, 100kb downstream of the gene, within the gene body), we considered two additional linear models that respectively included the presence of any germline SNVs (from dbSNP) and the presence of any somatic SVs within the same region window. As observed here, the top SV-expression associations are essentially the same with or without the germline SNV or somatic SV covariate. Of the three genes significant without germline SNV covariate at p<0.01 but not significant with the SNV covariate at p<0.01, two were significant with SNV covariate at p<0.02. Of the 17 genes significant without somatic SV covariate at p<0.01 but not significant with the somatic SV covariate at p<0.01, all were significant with somatic SV covariate at p<=0.02. In contrast to eQTLs, our analytical approach to identifying SV-expression association is region-specific rather than variant-specific. Here, we only consider whether any known SNVs are present within the same region as the SVs, consistent with our treatment of SVs in the models. **(B)** By tumor tissue of origin, enrichment patterns for a top set of 1493 recurrent SVs (p<0.00001 by chi-square for at least one tissue). Recurrent SVs represented were taken from the set of 5426 recurrent SVs represented in at least 20 patients. Unlike elsewhere (e.g., Figure 1C), no slop of 200 bp was used to define recurrent SVs. Only exact genomic coordinates were used here. **(C)** From Figure 2A, 317 genes had germline SV breakpoints 100kb upstream of the gene significantly associated with increased expression, with FDR<10% by linear modeling without correction for tumor tissue of origin. These 317 genes are represented here, separated by genes not significant after correction for tumor tissue type (p>0.01 by linear modeling) versus genes that were significant after tumor tissue type correction. Expression heat maps on the left represent the average differential expression by tumor tissue of origin. Heat maps on the right represent significant enrichment patterns, by tissue of origin, of germline SV breakpoints 100 kb upstream of the gene (p-values by one-sided Fisher's exact test). As opposed to the genes significant both with and without correction for tumor tissue of origin (represented in the bottom panels), most of the genes significant only for the model without tissue type correction (represented in the top panels) have both breakpoint enrichment pattern and higher average expression for at least one tissue type. For genes that lose significance when tissue type is incorporated into the model, the tissue type alone can explain the differential expression patterns in relation to SV breakpoints. **(D)** Similar to part B, but for the 330 genes with germline SV breakpoints 100kb downstream of the gene significantly associated with increased expression, with FDR<10% by linear modeling without correction for tumor tissue of origin. Related to Figure 2.

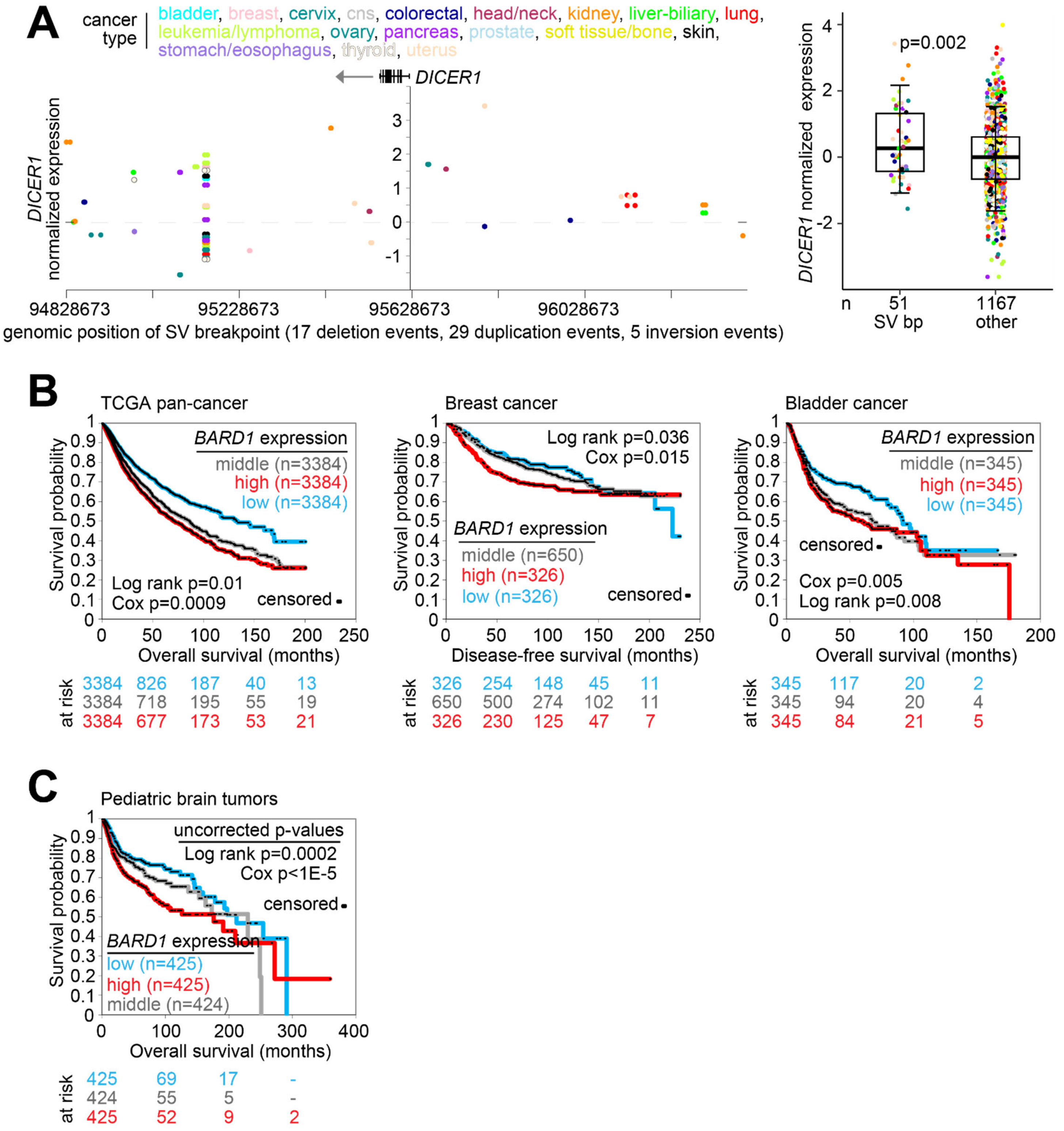
A
cancer type
bladder, breast, cervix, cns, colorectal, head/neck, kidney, liver-biliary, lung, leukemia/lymphoma, ovary, pancreas, prostate, soft tissue/bone, skin, stomach/eosophagus, thyroid, uterus
DICER1
DICER1 normalized expression
94828673
95228673
95628673
96028673
genomic position of SV breakpoint (17 deletion events, 29 duplication events, 5 inversion events)
p=0.002
DICER1 normalized expression
n
51
SV bp
1167
other
B
TCGA pan-cancer
BARD1 expression
middle (n=3384)
high (n=3384)
low (n=3384)
Log rank p=0.01
Cox p=0.0009
censored
Survival probability
Overall survival (months)
at risk
3384 826 187 40 13
3384 718 195 55 19
3384 677 173 53 21
Breast cancer
Log rank p=0.036
Cox p=0.015
BARD1 expression
middle (n=650)
high (n=326)
low (n=326)
censored
Disease-free survival (months)
at risk
326 254 148 45 11
650 500 274 102 11
326 230 125 47 7
Bladder cancer
BARD1 expression
middle (n=345)
high (n=345)
low (n=345)
censored
Cox p=0.005
Log rank p=0.008
Overall survival (months)
at risk
345 117 20 2
345 94 20 4
345 84 21 5
C
Pediatric brain tumors
uncorrected p-values
Log rank p=0.0002
Cox p<1E-5
censored
BARD1 expression
low (n=425)
high (n=425)
middle (n=424)
Survival probability
Overall survival (months)
at risk
425 69 17 -
424 55 5 -
425 52 9 2

**Figure S4. Additional information regarding germline SV breakpoint patterns involving essential genes in cancer cell lines. (A)** *DICER1* mRNA expression levels corresponding to germline SVs located in the genomic region ~787kb upstream or downstream of the gene (left). All SV breakpoints falling within the given region are represented. Boxplot (right, representing 5%, 25%, 50%, 75%, and 95%) shows *DICER1* expression by tumor samples with germline SV breakpoint downstream of the gene versus other tumors. Unlike the boxplots elsewhere for other genes featured in this study, normalized expression values for *DICER1* as shown here are normalized within each TCGA/ICGC project to standard deviations from the median, as unlike most other significant genes, DICER1 was not significant unless tumor tissue of origin was included as a covariate in the linear model, thereby normalizing for total expression differences by cancer type. P-value in boxplot by t-test on the normalized values. *DICER1* was significant across the 1Mb region surrounding the gene (which model weights the breakpoints by relative to the gene[6]) and for the 0-100kb upstream region, though the latter involved only two patients (Data File S3). Germline SV breakpoints further away than ~787kb from the gene did not contribute to the association with increased expression. The *DICER1*-associated SV breakpoints did not fall into a tight pattern of location as observed for the other genes of interest in this study. **(B)** Similar to Figure 6D, with Kaplan-Meier life tables included. Association of *BARD1* expression with worse patient outcome in TCGA pan-cancer (n=10,152 patients)[7], breast cancer (n=1302)[8], and bladder cancer (n=1035)[9] cohorts. P-values by log-rank test and by univariate Cox. For the TCGA pan-cancer dataset, tests correct for cancer type (by TCGA project). For breast dataset, survival is capped at 230 months. **(C)** Association of *BARD1* expression with worse patient outcome in pediatric brain tumor cohorts. P-values by log-rank test and by univariate Cox. Unlike for the Figure 7 KM plot involving the CBTN pediatric brain tumor dataset, p-values here are not corrected by histologic type. When incorporating histologic type as a variable, *BARD1* was not significant, indicating that it does not add any additional information in predicting outcome not already provided by histologic type. Related to Figure 6.

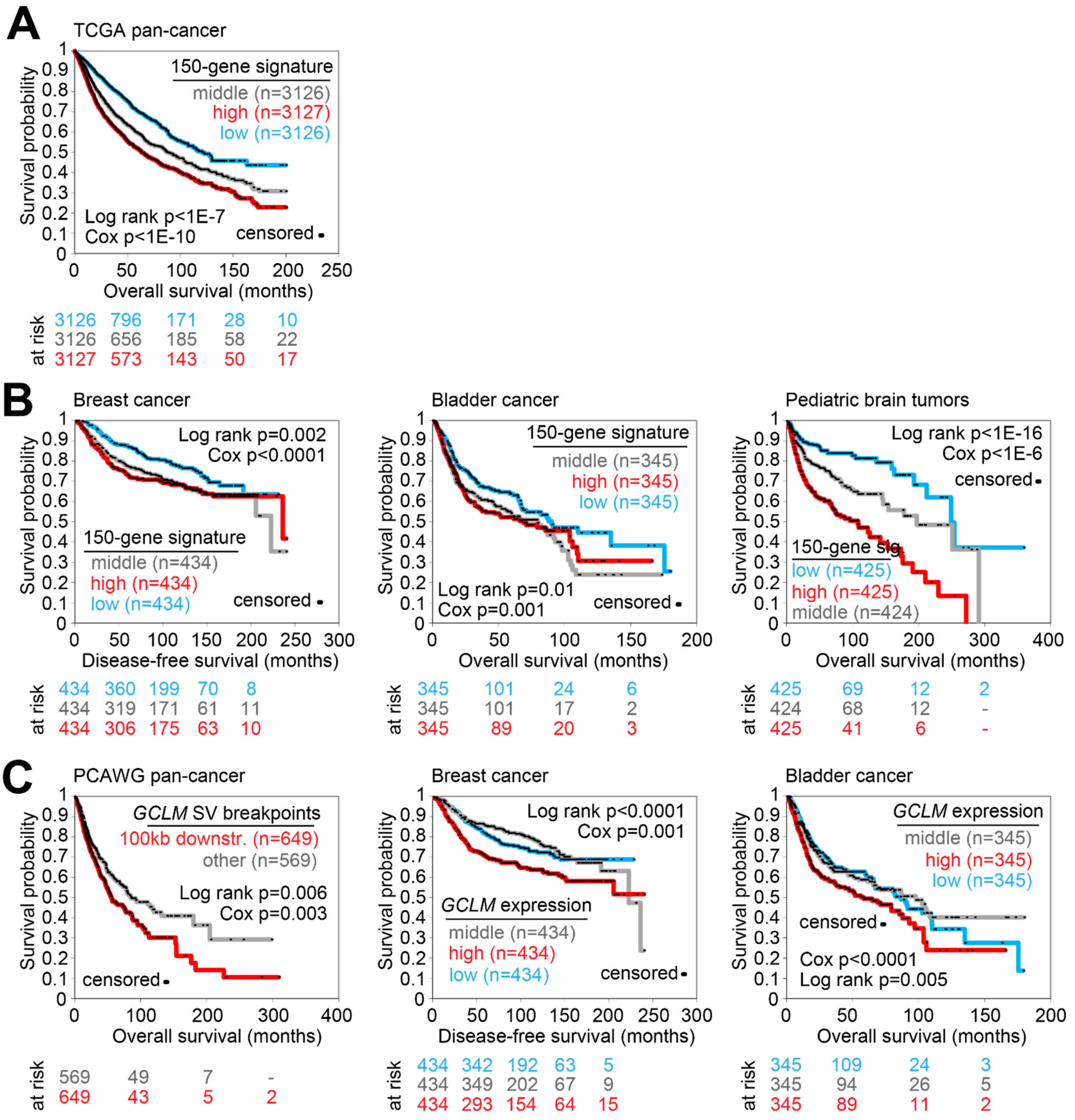
A
TCGA pan-cancer
150-gene signature
middle (n=3126)
high (n=3127)
low (n=3126)
Log rank p<1E-7
Cox p<1E-10
censored
Survival probability
Overall survival (months)
at risk
3126 796 171 28 10
3126 656 185 58 22
3127 573 143 50 17
B
Breast cancer
Log rank p=0.002
Cox p<0.0001
150-gene signature
middle (n=434)
high (n=434)
low (n=434)
censored
Disease-free survival (months)
at risk
434 360 199 70 8
434 319 171 61 11
434 306 175 63 10
Bladder cancer
150-gene signature
middle (n=345)
high (n=345)
low (n=345)
Log rank p=0.01
Cox p=0.001
censored
Overall survival (months)
at risk
345 101 24 6
345 101 17 2
345 89 20 3
Pediatric brain tumors
Log rank p<1E-16
Cox p<1E-6
censored
150-gene sig.
low (n=425)
high (n=425)
middle (n=424)
Overall survival (months)
at risk
425 69 12 2
424 68 12 -
425 41 6 -
C
PCAWG pan-cancer
GCLM SV breakpoints
100kb downstr. (n=649)
other (n=569)
Log rank p=0.006
Cox p=0.003
censored
Overall survival (months)
at risk
569 49 7 -
649 43 5 2
Breast cancer
Log rank p<0.0001
Cox p=0.001
GCLM expression
middle (n=434)
high (n=434)
low (n=434)
censored
Disease-free survival (months)
at risk
434 342 192 63 5
434 349 202 67 9
434 293 154 64 15
Bladder cancer
GCLM expression
middle (n=345)
high (n=345)
low (n=345)
censored
Cox p<0.0001
Log rank p=0.005
Overall survival (months)
at risk
345 109 24 3
345 94 26 5
345 89 11 2

**Figure S5. Additional information regarding germline SV breakpoint patterns involving cancer patient survival. (A)** Similar to Figure 7B, with Kaplan-Meier life tables included. Association of the 150-gene signature from Figure 7A with patient survival in TCGA pan-cancer dataset (n=9379, no PCAWG tumors)[7], based on scoring of the tumor expression profiles. The direction of each gene in the 150-gene signature, as applied here to the entire TCGA cohort, is based on the direction of the germline SV-expression association. As indicated, p-values by log-rank test and univariate Cox, corrected for cancer type. **(B)** Similar to Figure 7C, with Kaplan-Meier life tables included. Association of the 150-gene signature from part A with patient survival across multiple cancer types and three separate expression datasets: breast cancer (n=1302)[8], bladder cancer (n=1035)[9], and pediatric brain tumors (n=1274)[10]. P-values by log-rank test and by univariate Cox, as indicated. P-values corrected by histologic type for pediatric brain dataset. **(C)** Similar to Figure 7E, with Kaplan-Meier life tables included. Association of *GCLM* germline SV breakpoint patterns with worse patient outcome, and association of *GCLM* expression with worse patient outcome in breast and bladder cancer cohorts. P-values by log-rank test and by univariate Cox. For the SV breakpoints dataset, tests correct for tumor tissue of origin. For part A, Cox p-values are one-sided; all other Cox p-values in the figure are two-sided. Related to Figure 7.

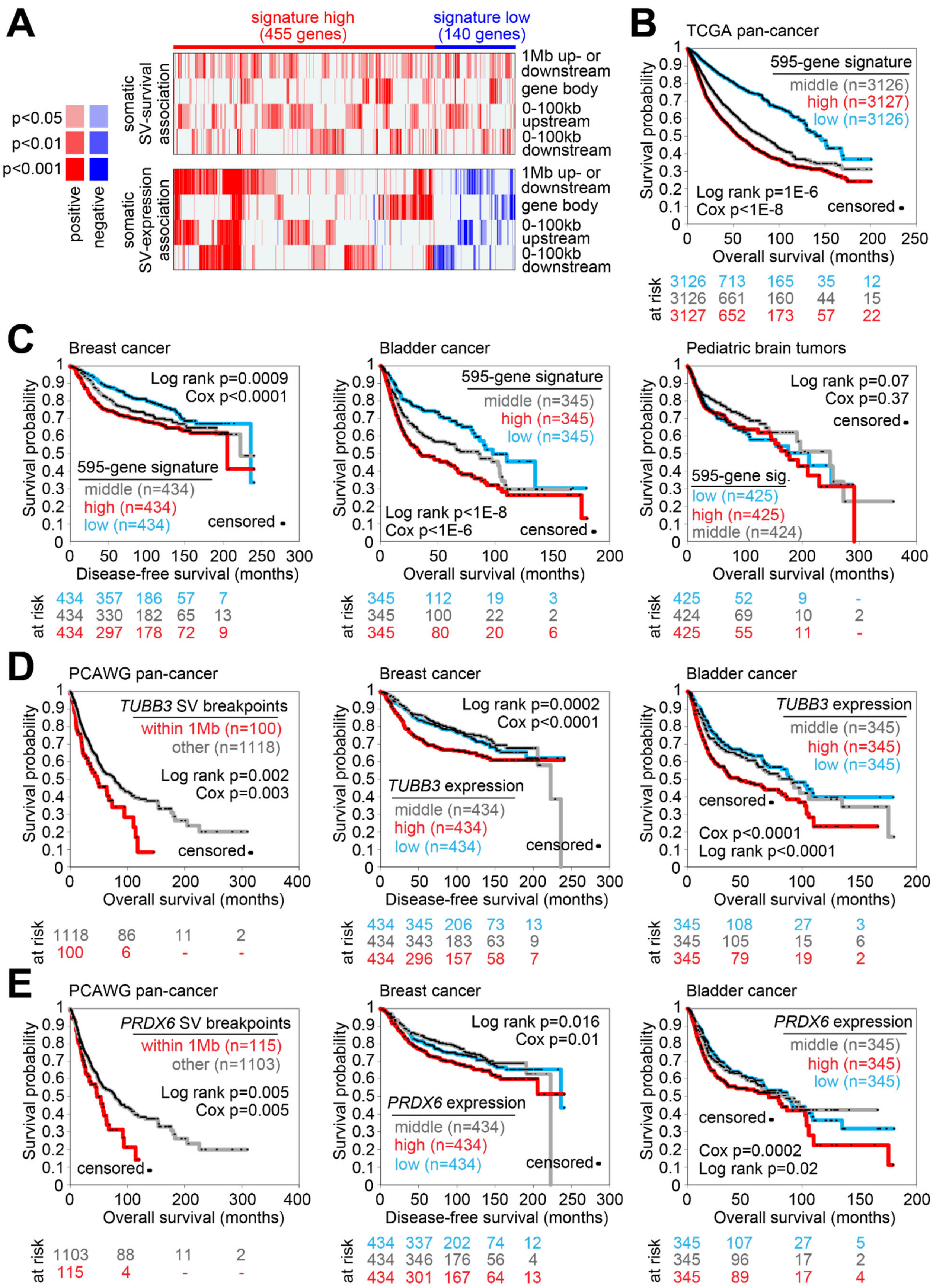
A
signature high (455 genes)
signature low (140 genes)
somatic SV-survival association
somatic SV-expression association
1Mb up- or downstream
gene body
0-100kb upstream
0-100kb downstream
p<0.05
p<0.01
p<0.001
positive
negative
B
TCGA pan-cancer
595-gene signature
middle (n=3126)
high (n=3127)
low (n=3126)
Log rank p=1E-6
Cox p<1E-8
censored
Survival probability
Overall survival (months)
at risk
3126 713 165 35 12
3126 661 160 44 15
3127 652 173 57 22
C
Breast cancer
Log rank p=0.0009
Cox p<0.0001
595-gene signature
middle (n=434)
high (n=434)
low (n=434)
censored
Disease-free survival (months)
434 357 186 57 7
434 330 182 65 13
434 297 178 72 9
Bladder cancer
595-gene signature
middle (n=345)
high (n=345)
low (n=345)
Log rank p<1E-8
Cox p<1E-6
censored
Overall survival (months)
345 112 19 3
345 100 22 2
345 80 20 6
Pediatric brain tumors
Log rank p=0.07
Cox p=0.37
censored
595-gene sig.
low (n=425)
high (n=425)
middle (n=424)
Overall survival (months)
425 52 9 -
424 69 10 2
425 55 11 -
D
PCAWG pan-cancer
TUBB3 SV breakpoints
within 1Mb (n=100)
other (n=1118)
Log rank p=0.002
Cox p=0.003
censored
Overall survival (months)
1118 86 11 2
100 6 - -
Breast cancer
Log rank p=0.0002
Cox p<0.0001
TUBB3 expression
middle (n=434)
high (n=434)
low (n=434)
censored
Disease-free survival (months)
434 345 206 73 13
434 343 183 63 9
434 296 157 58 7
Bladder cancer
TUBB3 expression
middle (n=345)
high (n=345)
low (n=345)
censored
Cox p<0.0001
Log rank p<0.0001
Overall survival (months)
345 108 27 3
345 105 15 6
345 79 19 2
E
PCAWG pan-cancer
PRDX6 SV breakpoints
within 1Mb (n=115)
other (n=1103)
Log rank p=0.005
Cox p=0.005
censored
Overall survival (months)
1103 88 11 2
115 4 - -
Breast cancer
Log rank p=0.016
Cox p=0.01
PRDX6 expression
middle (n=434)
high (n=434)
low (n=434)
censored
Disease-free survival (months)
434 337 202 74 12
434 346 176 56 4
434 301 167 64 13
Bladder cancer
PRDX6 expression
middle (n=345)
high (n=345)
low (n=345)
censored
Cox p=0.0002
Log rank p=0.02
Overall survival (months)
345 107 27 5
345 96 17 2
345 89 17 4

**Figure S6. Somatic SV breakpoint patterns involving cancer patient survival. (A)** Combining somatic SV data with patient survival data and tumor expression data across the 1218 PCAWG patients, 595 genes were identified, for which was found both a positive association between somatic SV breakpoints near the gene and worse overall survival and a positive or negative association between nearby somatic SV breakpoints and altered gene expression. Genes listed are significant for overall survival association of one-sided $p<0.05$ by univariate Cox (corrected for tissue type) for any genomic region window examined (100kb upstream of the gene, 100kb downstream of the gene, within the gene body, or 1Mb upstream or downstream of the gene), and significant for expression association for any genomic region window with $p<0.05$ by linear model correcting for tumor project, gene-level copy, tumor ploidy, and tumor purity. **(B)** Association of the 595-gene signature from part A with patient survival in TCGA pan-cancer dataset (n=9379, no PCAWG tumors)[7], based on scoring of the tumor expression profiles. The direction of each gene in the 595-gene signature, as applied here to the entire TCGA cohort, is based on the direction of the somatic SV-expression association. As indicated, p-values by log-rank test and univariate Cox, corrected for cancer type. **(C)** Association of the 595-gene signature from part A with patient survival across multiple cancer types and three separate expression datasets: breast cancer (n=1302)[8], bladder cancer (n=1035)[9], and pediatric brain tumors (n=1274)[10]. P-values by log-rank test and by univariate Cox, as indicated. **(D)** Association of *TUBB3* somatic SV breakpoint patterns with worse patient outcome, and association of *TUBB3* expression with worse patient outcome in breast cancer and bladder cancer cohorts. P-values by log-rank test and by univariate Cox. For the SV breakpoints dataset, Cox tests correct for tumor tissue of origin. **(E)** Similar to part D, but for *PRDX6* gene. For part A, Cox p-values are one-sided; all other Cox p-values in the figure are two-sided. Related to Figure 7.

## References


1. MacDonald, J., Ziman, R., Yuen, R., Feuk, L., and Scherer, S. (2014). The Database of Genomic Variants: a curated collection of structural variation in the human genome. Nucleic Acids Res *42*, D986-992.
2. Collins, R., Brand, H., Karczewski, K., Zhao, X., Alföldi, J., Francioli, L., Khera, A., Lowther, C., Gauthier, L., Wang, H., et al. (2020). A structural variation reference for medical and population genetics. Nature *581*, 444-451.
3. Mills, R., Walter, K., Stewart, C., Handsaker, R., Chen, K., Alkan, C., Abyzov, A., Yoon, S., Ye, K., Cheetham, R., et al. (2011). Mapping copy number variation by population-scale genome sequencing. Nature *470*, 59-65.
4. Scott, A., Chiang, C., and Hall, I. (2021). Structural variants are a major source of gene expression differences in humans and often affect multiple nearby genes. Genome Res *31*, 2249-2257.
5. Nattestad, M., Goodwin, S., Ng, K., Baslan, T., Sedlazeck, F., Rescheneder, P., Garvin, T., Fang, H., Gurtowski, J., Hutton, E., et al. (2018). Complex rearrangements and oncogene amplifications revealed by long-read DNA and RNA sequencing of a breast cancer cell line. Genome Res *28*, 1126-1135.
6. Zhang, Y., Yang, L., Kucherlapati, M., Hadjipanayis, A., Pantazi, A., Bristow, C., Lee, E., Mahadeshwar, H., Tang, J., Zhang, J., et al. (2019). Global impact of somatic structural variation on the DNA methylome of human cancers. Genome biology *20*, 209.
7. Chen, F., Zhang, Y., Gibbons, D., Deneen, B., Kwiatkowski, D., Ittmann, M., and Creighton, C. (2018). Pan-cancer molecular classes transcending tumor lineage across 32 cancer types, multiple data platforms, and over 10,000 cases. Clin Cancer Res. *24*, 2182-2193.
8. Creighton, C. (2012). The molecular profile of luminal B breast cancer. Biologics *6*, 289-297.
9. Chen, F., Zhang, Y., Chandrashekar, D., Varambally, S., and Creighton, C. (2023). Global impact of somatic structural variation on the cancer proteome. Nat Commun *14*, 5637.
10. Zhang, Y., Chen, F., Donehower, L., Scheurer, M., and Creighton, C. (2021). A pediatric brain tumor atlas of genes deregulated by somatic genomic rearrangement. Nat Commun *12*, 937.
